# Imaging small molecule-induced endosomal escape of siRNA

**DOI:** 10.1038/s41467-020-15300-1

**Published:** 2020-04-14

**Authors:** Hampus Du Rietz, Hampus Hedlund, Sten Wilhelmson, Pontus Nordenfelt, Anders Wittrup

**Affiliations:** 1 Department of Clinical Sciences, Oncology, Faculty of Medicine, Lund University, Lund, Sweden; 2 Wallenberg Center for Molecular Medicine, Lund, Sweden; 3 Skane University Hospital, Lund, Sweden; 4 Department of Clinical Sciences, Division of Infection Medicine, Faculty of Medicine, Lund University, Lund, Sweden

**Keywords:** RNAi therapy, Oligo delivery, Cellular imaging

## Abstract

Small interfering RNAs (siRNAs) are a new class of promising therapeutic molecules that can be used for sequence-specific downregulation of disease-causing genes. However, endosomal entrapment of siRNA is a key hurdle for most delivery strategies, limiting the therapeutic effect. Here, we use live-cell microscopy and cytosolic galectin-9 as a sensor of membrane damage, to probe fundamental properties of endosomal escape of cholesterol-conjugated siRNA induced by endosome-disrupting compounds. We demonstrate efficient release of ligand-conjugated siRNA from vesicles damaged by small molecules, enhancing target knockdown up to ∼47-fold in tumor cells. Still, mismatch between siRNA-containing and drug-targeted endolysosomal compartments limits siRNA activity improvement. We also show widespread endosomal damage in macroscopic tumor spheroids after small molecule treatment, substantially improving siRNA delivery and knockdown throughout the spheroid. We believe the strategy to characterize endosomal escape presented here will be widely applicable, facilitating efforts to improve delivery of siRNA and other nucleic acid-based therapeutics.

## Abstract (web summary)

Therapeutic siRNA becomes trapped in endosomes, limiting its efficacy. Here the authors use fluorescently-tagged galectin-9 as a biosensor for membrane damage to monitor endosomal escape of cholesterol-conjugated siRNA following treatment of small molecule membrane-destabilising drugs.

## Introduction

Small interfering RNAs (siRNA) are double-stranded RNA molecules, 21–23 nucleotides long, which can downregulate the expression of practically any gene. Therapeutics based on siRNA offer the prospect to open up a vast array of novel drug targets for pharmacological inhibition. However, efficient delivery of macromolecular siRNA to the cytosol of target cells is challenging. Diverse delivery strategies have been explored, including viruses^1,2^, lipid nanoparticles (LNPs)^3^, and ligand-conjugated siRNAs^4,5^.

Patisiran, an LNP-formulated siRNA targeting transthyretin (TTR) in the liver, was recently the first siRNA therapeutic to be approved for clinical use. LNPs preferentially target the liver, where the fenestrated endothelium enables extravasation and endocytosis by hepatic cells^6,7^. How siRNA exits the endosome and enters the cytosol, a process referred to as endosomal escape, has long been poorly understood. Lately, progress has been made in the elucidation of lipid-mediated endosomal escape of siRNA. Electron microscopy studies of liver tissues suggested that LNPs mediate siRNA release in the early stages of the endosomal system^8^. In addition, an endogenous family of β-galactoside-binding lectins—galectins—were shown to respond to endosomal membrane disruption and rapidly relocate to damaged vesicles during lipid-mediated endosomal escape, making detailed characterization of the process possible^9^. Lipids induce release of the siRNA payload in a narrow “window of opportunity” during the endosomal maturation process, after formation of early endosomes but before the siRNA reaches mature lysosomes, characterized by the lysosome-associated membrane protein 1 (LAMP1)^9^.

Efficient targeting of LNPs to extra-hepatic tissues has turned out to be challenging. To reach tissues without fenestrated endothelium, and in particular tumors, non-particulate siRNA formulations would be advantageous. Chemically stabilized free siRNA molecules, covalently attached to targeting ligands, can be used to efficiently traffic siRNA to specific tissues and tumors^4,10–12^. However, the vast majority of the internalized molecules are sequestered in the endosomal system, unable to reach the cytosol and the RNA interference (RNAi) machinery^12^. This can be partially overcome by targeting highly abundant cell surface receptors. One notable example is the asialoglycoprotein receptor (ASGPR) expressed by hepatocytes, which mediates highly efficient internalization of multivalent *N*-acetyl galactosamine- (GalNAc) conjugated siRNA^10^. A single subcutaneous injection of GalNAc-siRNA results in more than 80% target gene knockdown in human livers for longer than 180 days^13^. However, no additional ligand–receptor pair of equal potency has been found, and ASGPR expression is largely restricted to the liver. Achieving meaningful knockdown in other tissues or tumors will require substantially higher delivery efficiency than what is currently accomplished.

Cholesterol-conjugated chemically stabilized siRNA has been shown to accumulate in solid tumors^14,15^. At high doses, particularly when delivered locally, cholesterol-conjugated siRNA (chol-siRNA) induces significant knockdown^16^. As with other ligand-conjugated siRNAs, the high doses required to produce meaningful knockdown both in vitro and in vivo—despite pronounced siRNA accumulation in intracellular vesicles after uptake—indicates limited endosomal escape is the major efficiency-limiting factor. While release-enhancing strategies using endosmolytic polymers or peptides have been promising^17,18^, such approaches are challenging from a formulation, toxicity, and bioavailability perspective. Efforts have also been made to identify small-molecule drugs capable of enhancing oligonucleotide delivery^19–22^. However, direct visualization or analysis of enhanced endosomal escape of non-formulated oligonucleotides has not been possible. Consequently, it has not been clear to what extent the enhanced biological activity observed with such small molecules were due to improved endosomal escape of siRNA^23^.

Here, we speculated that small molecules with known membrane-destabilizing properties might facilitate endosomal escape of chol-siRNA. From existing literature^24^, we selected three small-molecule drugs: chloroquine, an antimalarial therapeutic widely studied pre-clinically for its effects on the endolysosomal system; siramesine, a σ-2 receptor agonist; and the tricyclic antidepressant amitriptyline. Together with a large number of additional small molecules, these are classified as cationic amphiphilic drugs (CADs) and collectively considered to accumulate in acidic compartments, provoking damages to lysosomes and potentially other intracellular membranes through various mechanisms^24–27^.

We have established a widely applicable imaging strategy, based on the sensitive membrane damage sensor galectin-9, to detect vesicle disruption triggered by these small molecules, and probe fundamental properties of endosomal escape facilitated by them. Our work reveals that membrane-destabilizing drugs are diverse and target multiple intracellular compartments. Drug-induced disruption of siRNA-containing vesicles results in efficient endosomal escape of chol-siRNA, substantially increasing knockdown efficiency. However, mismatch between siRNA-containing and drug-targeted compartments limits the siRNA activity improvement.

## Results

### Galectin-9 is a sensor of drug-induced membrane disruption

**Fig. 1:**
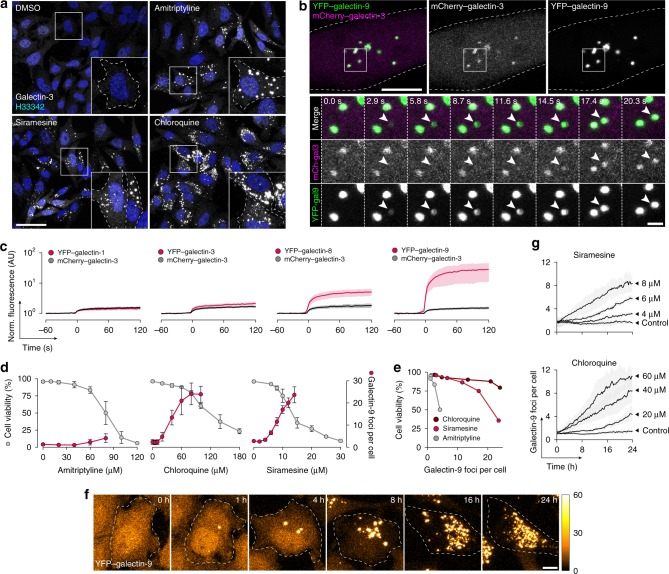
Galectin-9 is a reliable sensor of drug-induced membrane perturbation in live cells. **a** Confocal microscopy images of HeLa cells stained for galectin-3 after treatment with 10 μM siramesine, 50 μM amitriptyline, or 50 μM chloroquine for 24 h. Images are representative of two independent experiments. Scale bar, 50 μm. **b** HeLa cells co-expressing mCherry–galectin-3 and YFP–galectin-9 were imaged with confocal microscopy during treatment with 10 μM siramesine. Image subset shows de novo galectin recruitment. Images are representative of four independent experiments. Scale bar, 10 μm; detail 2 μm. **c** Quantification of galectin recruitment from confocal microscopy images of HeLa cells expressing the indicated galectin pairs, treated with 10 μM siramesine. Line is mean, shade is 95% CI. Traces are aligned so that galectin recruitment is first detected at *t* = 0. *N* = 52, 54, 45, and 54 events from at least two independent experiments. **d** Cell viability after 24 h drug treatment was determined by propidium iodide staining and flow cytometry. The number of galectin-9 foci per cell was quantified from confocal microscopy images after 24 h drug treatment. Mean ± s.d. from three independent experiments. **e** Effect of increasing number of galectin-9 foci on viability. Mean values from **d**. **f** HeLa cells expressing YFP–galectin-9 was monitored by confocal microscopy during treatment with 60 μM chloroquine. Images are representative of three independent experiments. Scale bar, 10 μm. **g** Number of galectin-9 foci per cell quantified from confocal microscopy images acquired during 24 h drug treatment as indicated. Line is mean, shade is s.d. *N* = three independent experiments. Source data for **c**–**e**, **g** are presented in the Source Data File.

To enable detailed investigation of drug-induced endosomal escape, we first evaluated members of the galectin family in live cells as sensors of vesicle damage caused by membrane-destabilizing drugs. Consistent with previous reports^28^, staining HeLa cells with anti-galectin-3 after 24 h incubation with amitriptyline, siramesine, or chloroquine revealed the formation of intracellular galectin foci, as evidence of membrane damages (Fig. 1a). Galectin-3 foci were scarce in untreated control cells.

Other members of the galectin family are also known to be recruited to vesicles damaged by invading bacteria, viruses, and osmotic shock^29,30^. Galectin-1, -3, -8, and -9 were most abundantly recruited to endocytic vesicles damaged by transfection lipids^9^. It is not clear if the galectins have distinct compartment specificity, or different sensitivity to detect membrane disruption in live cells caused by small-molecule compounds. To elucidate this, HeLa cells co-expressing mCherry–galectin-3 as reference, and one of the YFP-tagged galectin-1, -3, -8, and -9 were treated with siramesine to trigger membrane damage during live-cell microscopy. Recruitment of galectin-9 was both most abundant and rapid, with a signal increase in the targeted structure ∼14-fold higher than with galectin-3 (Fig. 1b, c). Typically, galectin-9 recruitment was fast enough to pinpoint the time of the event to the exact frame, which was often not possible using galectin-3. In contrast to galectin-3, galectin-9 foci were also detectable in the nuclei, both in small-molecule-treated and control cells. These foci were static and appeared stationary within the nucleus. Thus, the intranuclear galectin-9 foci likely represents a process separate from the recognition of damaged cytosolic vesicles, distinguishable as de novo formed galectin foci.

Regarding compartment specificity, both galectin-3 and -9 detect all damages to the endolysosomal system caused by transfection agents^9^. Here, using a small-molecule drug, all de novo formed galectin-3^+^ structures were galectin-9^+^, while a few faint galectin-9 foci were negative for galectin-3 (∼6%, *N* = 52 events) (Supplementary Fig. 1). Thus, as a sensor of membrane damage caused by small molecules in live cells, galectin-9 has the fastest detection kinetics, highest detection sensitivity and possibly the broadest compartment specificity among the galectin family members evaluated.

Lysosomal membrane permeabilization and formation of galectin foci is a known step during lysosome-induced cell death triggered by CADs^31^. However, with both siramesine and chloroquine, numerous galectin-9 foci were observable at drug concentrations well below significant toxicity (Fig. 1d). Viability remained high up to 15–20 galectin-9 foci per cell, in particular with chloroquine. For amitriptyline, toxicity was higher at doses generating the same number of galectin foci (Fig. 1e). During time-lapse microscopy, gradual emergence of galectin-9 foci with maintained viability (and proliferation) over many hours was also observed (Fig. 1f, g and Supplementary Movie 1). This suggests that the toxicity seen at higher concentrations is not solely a consequence of increasing numbers of damaged vesicles. Instead, other drug-specific toxic effects contribute.

### Membrane destabilization improves siRNA-mediated knockdown

**Fig. 2:**
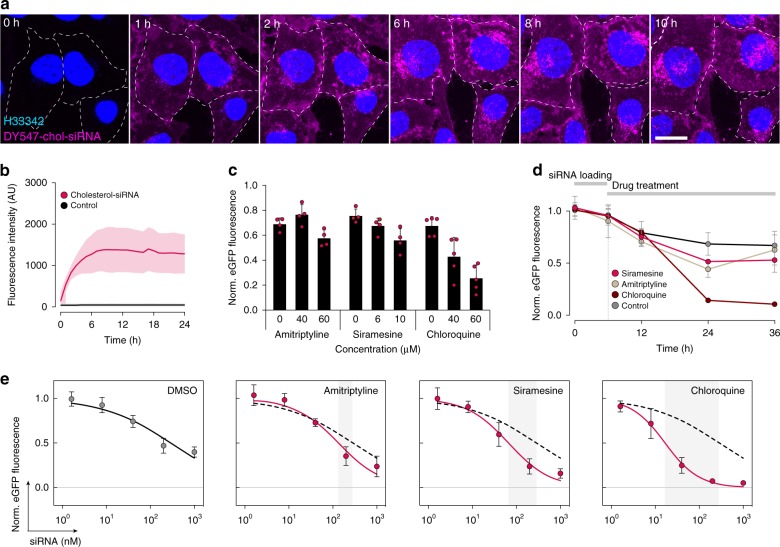
Membrane-destabilizing drugs improve chol-siRNA-mediated knockdown. **a** Chol-siRNA internalization was monitored for 24 h in HeLa cells using confocal microscopy. Representative images from two independent experiments. Scale bar, 20 μm. **b** Quantification of chol-siRNA internalization from confocal microscopy images. Lines are means, shades are s.d. *N* = 3 independent experiments. **c** HeLa-d1-eGFP cells were incubated with 40 nM chol-siGFP for 6 h, and treated with amitriptyline, siramesine, or chloroquine at the concentrations indicated for 18 h, or **d** 10 μM siramesine, 60 μM amitriptyline, or 60 μM chloroquine for up to 30 h. eGFP knockdown was determined by flow cytometry. Mean ± s.d. is shown. *N* = 4 (amitriptyline, siramesine), 5 (chloroquine) independent experiments (**c**), and *N* = 3 independent experiments (**d**). **e** HeLa-d1-eGFP cells were incubated with the indicated concentrations of chol-siGFP for 6 h, followed by treatment with 10 μM siramesine, 60 μM amitriptyline, or 60 μM chloroquine for 18 h. eGFP knockdown was determined by flow cytometry. Circles are mean, and bars are s.d. *N* = 7, 5, 5, and 6 independent experiments. Dashed lines indicate DMSO control. Gray areas indicate IC_50_ shifts. Source data for **b**–**e** are presented in the Source Data file.

**Fig. 3:**
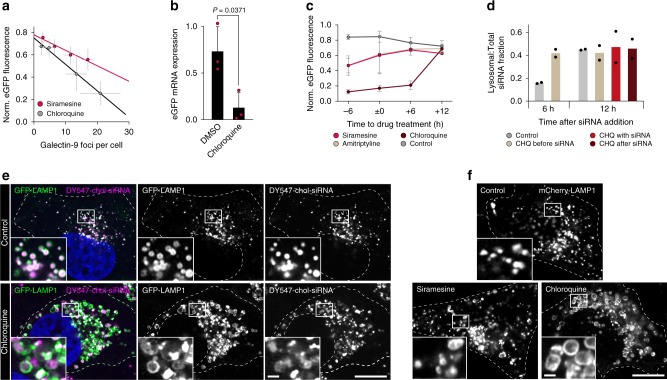
Knockdown is improved by matching siRNA delivery and small-molecule effects. **a** Effect on eGFP fluorescence intensity by increasing number of galectin-9 foci. Mean ± s.d. from Figs. 1d and 2e. *N* = 3 (galectin-9 foci quantification), 4 (siramesine eGFP knockdown), 5 (chloroquine eGFP knockdown) independent experiments. Statistical testing was performed by linear regression. **b** HeLa-d1-eGFP cells were incubated with 40 nM chol-siGFP for 6 h, followed by 60 μM chloroquine for 18 h. eGFP knockdown was determined by real-time quantitative PCR. Mean ± s.d. from three independent experiments. Two-tailed Student’s *t* test. **c** HeLa-d1-eGFP cells were treated with 60 μM chloroquine, 60 μM amitriptyline, or 10 μM siramesine starting 6 h before, at the same time, or 6 or 12 h after incubation with 40 nM chol-siGFP for 6 h. eGFP knockdown was determined by flow cytometry 18 h after starting siRNA incubation. Mean ± s.d. is shown. *N* = 3 independent experiments. **d** HeLa cells expressing GFP-LAMP1 were treated with 60 μM chloroquine (CHQ) starting 6 h before, at the same time or 6 h after incubation with 200 nM DY547-chol-siRNA for 6 h. Cells were fixed 6 or 12 h after siRNA addition and imaged with an Airyscan confocal microscope. The fraction of chol-siRNA fluorescence intensity colocalizing with GFP-LAMP1 objects are shown. Bars are mean. *N* = two independent experiments, each with 10 cells per condition. **e**, **f** Airyscan confocal microscopy images of HeLa cells expressing GFP-LAMP1 or mCherry-LAMP1, **e** after incubation with 200 nM DY547-chol-siRNA for 6 h, followed by 6 h treatment with 60 μM chloroquine or no treatment, or **f** after incubation with 10 μM siramesine or 60 μM chloroquine only for 12 h. Representative images from two independent experiments, each with 10 cells per condition. Scale bars, 10 μm; details, 1 μm. Source data for **a**–**d** are presented in a Source Data file.

We next investigated if drug-induced vesicle disruption could be used to enhance the delivery of chol-siRNA. Chol-siRNA is efficiently internalized by tumor cells in vitro and in vivo, but largely trapped in endosomal structures due to inefficient endosomal escape. When incubating HeLa cells with chol-siRNA, most uptake occurred during the first 6 h, with little additional intracellular accumulation thereafter (Fig. 2a, b). To evaluate the effect of membrane-disrupting drugs on siRNA-mediated knockdown, HeLa cells expressing a destabilized enhanced green fluorescent protein (d1-eGFP, *t*_1/2_ ∼1 h^32^) were incubated with chol-siRNA targeting eGFP (chol-siGFP) for 6 h, before initiating drug treatment. Chloroquine and to a lesser extent siramesine and amitriptyline enhanced chol-siGFP knockdown in a dose-dependent manner (Fig. 2c). Knockdown enhancement increased over time and was most pronounced after >24 h treatment (Fig. 2d), consistent with the gradual appearance of endosomal damages. The half-maximal inhibitory concentration (IC_50_) of chol-siGFP was only marginally reduced with amitriptyline and siramesine, (∼2- and 4-fold, respectively) (Fig. 2e). In contrast, chloroquine lowered chol-siGFP IC_50_ ∼17-fold (from 289 to 17 nM). For both chloroquine and siramesine, the knockdown improvement was associated with the number of galectin foci per cell (*P* = 0.0045 and 0.023, respectively, two-tailed Student’s *t* test) (Fig. 3a). Interestingly, similar numbers of membrane damages resulted in a higher degree of knockdown enhancement with chloroquine compared to siramesine (linear regression, slopes: −0.024 and −0.014, *P* = 0.0223). Chloroquine-mediated knockdown improvement was further corroborated at the messenger RNA (mRNA) level with quantitative PCR (qPCR) (Fig. 3b). Thus, in particular chloroquine-induced endosome disruption potentiates the biological activity of chol-siGFP, suggesting improved cytosolic siRNA delivery.

We next wanted to determine if the enhanced d1-eGFP knockdown was dependent on the timing between siRNA administration and start of small-molecule treatment. HeLa-d1-eGFP cells were treated with amitriptyline, siramesine, or chloroquine before, after, or at the same time as initiating a 6-h chol-siGFP incubation. Earlier start of drug treatment was generally more effective, possibly due to a longer period with exposure to damaged endosomes given the delay of the vesicle-disrupting effect (Fig. 3c). Surprisingly, delaying small-molecule treatment until 12 h after initiating siRNA incubation completely abrogated knockdown enhancement for all three drugs. Chol-siRNA was internalized efficiently and localized to structures marked by LAMP1, irrespective of the timing of chloroquine treatment (Fig. 3d, e). Notably, LAMP1^+^ vesicles were substantially enlarged and deformed by drug treatment, in particular with chloroquine (Fig. 3f). Taken together, this suggests there is a broad time window around the moment of chol-siRNA administration, during which co-administration of an endosome-disrupting agent can improve target knockdown.

### Drug-induced release of dextran from endolysosomal vesicles

While triggering similar numbers of endosomal damages, different small molecules evidently vary in their ability to enhance siRNA-mediated knockdown. This can potentially be explained by multiple factors, including differences in compartment specificity, size or properties of the formed pores, effects on uptake, intracellular sorting, or degradation of the siRNA. Small-molecule drugs could also have effects unrelated to the delivery process, for example, direct effects on the RNAi machinery.

**Fig. 4:**
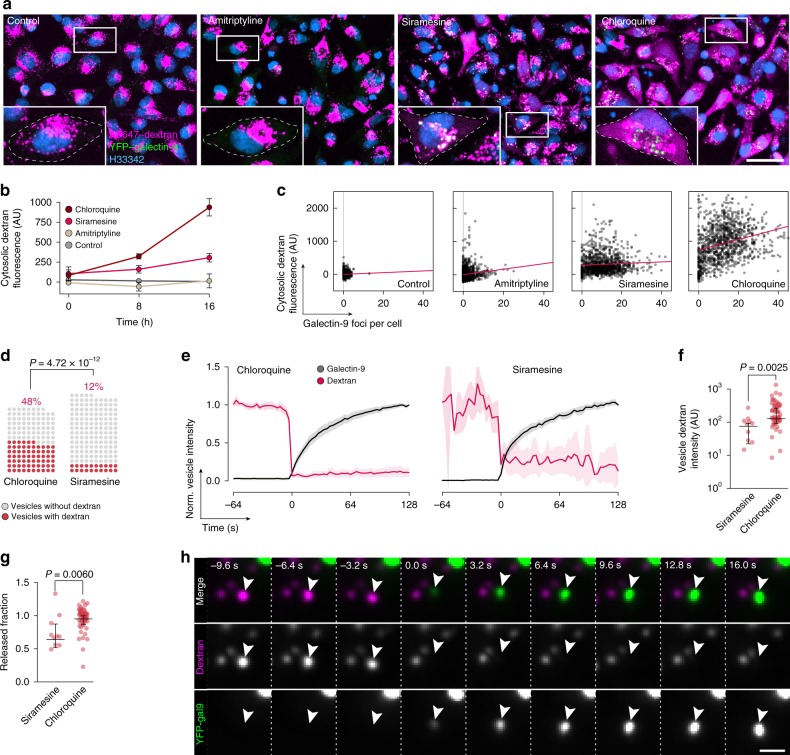
Linking single-vesicle release and cytosolic dispersion of dextran. **a** Confocal microscopy images of HeLa cells expressing YFP–galectin-9 incubated with 200 μg mL^−^^1^ AF647-dextran for 4 h followed by 2 h chase, and treated with 10 μM siramesine, 60 μM amitriptyline, or 60 μM chloroquine for 16 h. Images are representative details from three independent experiments. Scale bar, 40 μm. **b** Quantification of cytosolic dextran fluorescence intensity from confocal microscopy images in **a**. Mean ± s.d. from three independent experiments. **c** Number of galectin-9 foci and median cytosolic dextran fluorescence intensity per cell after 16 h drug treatment. Three independent experiments. *N* = 953, 1204, 1177, and 1139 cells. Red line is linear regression. **d**–**h** Live-cell widefield microscopy was used to monitor HeLa cells expressing YFP–galectin-9 incubated with AF647-dextran for 4 h, followed by 2 h chase before treatment with 60 μM chloroquine or 10 μM siramesine. **d** Fraction of damaged endosomes containing dextran before galectin-9 recruitment. *N* = 138 and 165 vesicles from two independent experiments. Two-tailed Fisher’s exact test. **e** Single-vesicle tracking was used to quantify dextran release from damaged endosomes. Lines are means, shades are 95% CI. Traces are aligned so that galectin recruitment is first detected at *t* = 0. *N* = 54 and 10 vesicles from two independent experiments. **f** Dextran fluorescence intensities of releasing endosomes measured ∼15 s before detectable galectin-9 recruitment, and **g** fraction of dextran released from single endosomes calculated from vesicle intensities ∼15 s before and ∼45 s after detectable galectin-9 recruitment. Median ± interquartile range. *N* = 10 and 54 vesicles from two independent experiments. Two-tailed Mann–Whitney test. **h** Arrows indicate release of dextran from an endosome showing galectin-9 recruitment during chloroquine treatment. Images are representative of 66 release events from two independent experiments. *t* = 0 is the first frame with detectable galectin recruitment. Scale bar, 2 μm. Source data for **b**–**g** are presented in a Source Data file.

Previously, it was shown that membrane-destabilizing drugs can trigger cytosolic release of inert macromolecules like dextran from endolysosomal compartments^33^. In contrast to siRNAs, which are actively redistributed after release from endosomes (Hedlund et al., unpublished observation, 2020), dextran remains homogeneously distributed in the cytoplasm for an extended period. This facilitates detection of macromolecules escaping from damaged vesicles. Accordingly, to address the differences seen between chloroquine and siramesine with respect to knockdown improvement, we asked if the drugs varied in their ability to induce cytosolic release of 10 kDa fluorescent dextran. In untreated control cells, internalized dextran was located in intracellular vesicles, and very few cells had a cytosolic dextran distribution (Fig. 4a). The majority of cells incubated with siramesine had detectable, but low levels of redistributed dextran after 16 h treatment. In contrast, pronounced cytosolic redistribution was apparent in cells treated with chloroquine at this time point (Fig. 4a, b). The amount of dextran released into the cytosol was associated with the number of detectable galectin foci in each cell after chloroquine treatment (Fig. 4c), while this effect was much weaker with siramesine (linear regression with 95% confidence interval [CI]: chloroquine, 16.1 (13.0–19.2); siramesine, 2.45 (0.16–4.74)). Amitriptyline treatment provoked fewer membrane damages and very limited cytosolic redistribution of dextran. Thus, with similar numbers of damaged vesicles, chloroquine treatment releases more inert macromolecules from endosomes compared to siramesine.

The observed differences in dextran release efficiency of siramesine and chloroquine could be due to (i) dextran-containing vesicles being targeted with different frequency, (ii) vesicles targeted having higher or lower dextran content, or (iii) differences in the fraction of dextran being released following vesicle damage. To investigate the process of cargo release from damaged endosomes in detail, we used live-cell widefield deconvolution microscopy, which provide suitable spatiotemporal resolution with low-intensity illumination. Importantly, given that damage events were relatively scarce, we were able to sample almost the entire cell volume for typically 500 time points with no signs of phototoxicity. During chloroquine treatment, ∼45% of damaged vesicles contained dextran just prior to galectin-9 recruitment (Fig. 4d). With siramesine, only ∼10% of damaged vesicles had detectable dextran content. Dextran release was fast upon vesicle damage (Fig. 4e), typically resulting in complete loss of dextran signal within the ∼3-s temporal resolution of the acquisitions. The dextran content of vesicles damaged during chloroquine treatment was higher than for siramesine (Fig. 4f). In the vast majority of cases, no or only minute amounts of dextran were detectable in the vesicle remnant (Fig. 4g). Release detection and evaluation were aided by rapid recruitment of galectin-9, typically apparent within 1–2 frames (∼3–6 s) from dextran release (Fig. 4h) The vast majority of dextran-containing vesicles colocalized with LAMP1 when release and galectin-9 recruitment was observed (Supplementary Fig. 2). Taken together, the more efficient cumulative dextran release seen with chloroquine treatment compared to siramesine is due to both higher hit-rate of dextran-containing vesicles, and targeting of vesicles with overall higher dextran content.

### Endosomal release of siRNA is facilitated by small molecules

**Fig. 5:**
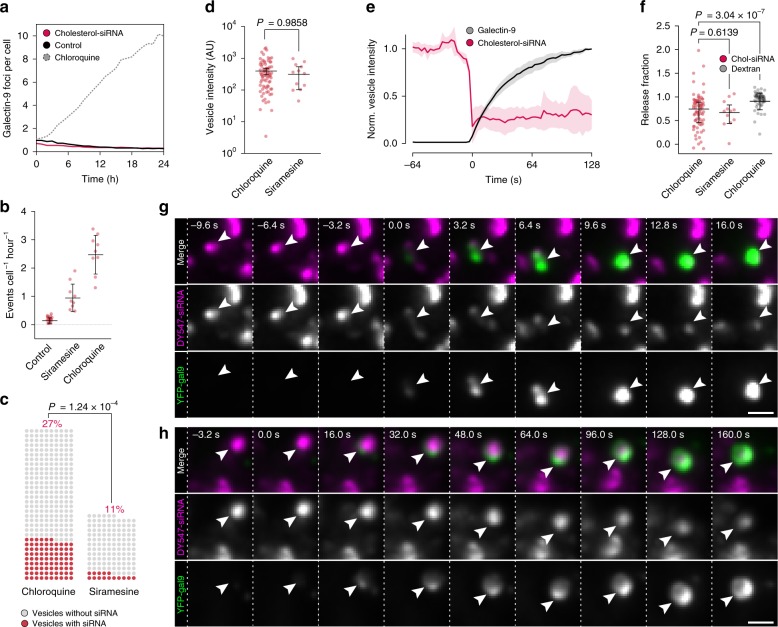
Endosomal release of chol-siRNA is facilitated by small molecules. **a** The number of galectin-9 foci per cell was monitored using confocal microscopy during 24 h incubation with 200 nM chol-siRNA. At least 359 control and 737 siRNA-treated cells were analyzed per time point, from two independent experiments. Lines are mean of two experiments. Dotted line is 40 μM chloroquine for reference. **b**–**h** HeLa cells expressing YFP–galectin-9 were incubated with 200 nM chol-siRNA for 6 h, followed by treatment with 60 μM chloroquine or 10 μM siramesine, while acquiring images with widefield microscopy. **b** The mean rate of galectin-9 foci formation was calculated in individual microscopy acquisitions. Bars indicate mean ± s.d. *N* = 21, 9, and 9 time-lapse acquisitions. Cells observed and mean observation time given as *N* (min) = 657 (27), 283 (25), and 294 (27). **c** Fraction of damaged endosomes containing chol-siRNA before galectin-9 recruitment. *N* = 320 and 136 vesicles from two (chloroquine) or three (siramesine) independent experiments. Two-tailed Fisher’s exact test. **d** Chol-siRNA fluorescence intensities of disrupted endosomes measured ∼15 s before detectable galectin-9 recruitment. Median ± interquartile range. *N* = 78 and 12 vesicles from two (chloroquine) or three (siramesine) independent experiments. **e** Quantification of chol-siRNA release during chloroquine treatment by single-vesicle tracking. Line is mean, shade is 95% CI. Traces are aligned so that galectin recruitment is first detected at *t* = 0. *N* = 78 vesicles from two independent experiments. **f** Fraction of chol-siRNA or dextran released from single endosomes calculated from vesicle intensities ∼15 s before and ∼45 s after detectable galectin-9 recruitment. Median ± interquartile range. *N* = 78, 12, and 54 vesicles from two, three, or two independent experiments, respectively. Two-tailed Mann–Whitney test. **g**, **h** Live-cell widefield microscopy images showing galectin-9 recruitment to endosomes containing DY547-chol-siRNA during chloroquine treatment. Arrows indicate vesicles with galectin-9 recruitment and siRNA release. Images are representative of 78 release events from two independent experiments. *t* = 0 is the first frame with detectable galectin recruitment. Scale bar, 2 μm. Source data for **a**–**f** are presented in a Source Data file.

Without the addition of any small-molecule drug, the number of detectable galectin-9 foci did not increase during continuous incubation with chol-siRNA for 24 h (Fig. 5a), indicating that chol-siRNA does not induce membrane damages on its own. To detect release of chol-siRNA induced by small molecules, we incubated cells expressing YFP–galectin-9 with DY547-labeled chol-siRNA for 6 h, before starting treatment with either siramesine or chloroquine. The rate of de novo galectin-9 foci formation with no small molecule present was low, but increased up to 30-fold, to ∼1–3 events per cell and hour, during drug treatment (Fig. 5b). With siramesine treatment, damaged vesicles contained chol-siRNA in 11% of cases, compared to 27% with chloroquine (Fig. 5c). No substantial difference was observed with respect to the amount of siRNA contained in vesicles damaged during chloroquine and siramesine treatment (Fig. 5d). As observed with dextran, a sudden decrease of the siRNA fluorescence intensity of the damaged vesicle typically occurred immediately before galectin recruitment was detectable (Fig. 5e, Supplementary Fig. 3a, and Supplementary Movie 2). In contrast to dextran release, however, a variable residual amount of siRNA was often present after release (Fig. 5f), with a fraction of events showing considerably more inefficient or prolonged release dynamics (rapid release: Fig. 5g and Supplementary Fig. 3b; slow release: Fig. 5h). The release efficiency did not depend on the extent of galectin-9 recruitment (Supplementary Fig. 3c). By manual classification, we observed that chol-siRNA was consistently released from LAMP1^+^ vesicles during chloroquine treatment (16 out of 16 release events, *N* = 53 damage events, Supplementary Fig. 3d, e and Supplementary Movie 3). Thus, membrane damages induced by both siramesine and chloroquine permit efficient chol-siRNA release from disrupted vesicles, but chloroquine targets vesicles containing siRNA to a significantly larger extent.

### Mapping compartment specificity of membrane-damaging drugs

The difference between chloroquine and siramesine with respect to dextran and chol-siRNA release efficiency prompted us to investigate the identity of the compartments damaged by these drugs. In previous studies, both chloroquine and siramesine have been suggested to primarily damage lysosomes^28,34^. However, detailed mapping of disrupted vesicles, particularly at the actual time of endosome damage, have not been reported. Thus, we aimed to assess the compartment characteristics of individual vesicles as close to the damage event as possible, by expressing markers of endosomal compartments together with YFP–galectin-9 in HeLa cells. Live-cell imaging enabled us to detect de novo recruitment of galectin-9 during siramesine or chloroquine treatment with ∼3-s temporal resolution. Objects marked by galectin-9 were tracked in 3D, quantifying the presence of the endosomal marker on the damaged structures. Measurements used for analysis were limited to the first ∼20 s after detectable galectin recruitment, that is, the moment of membrane damage.

**Fig. 6:**
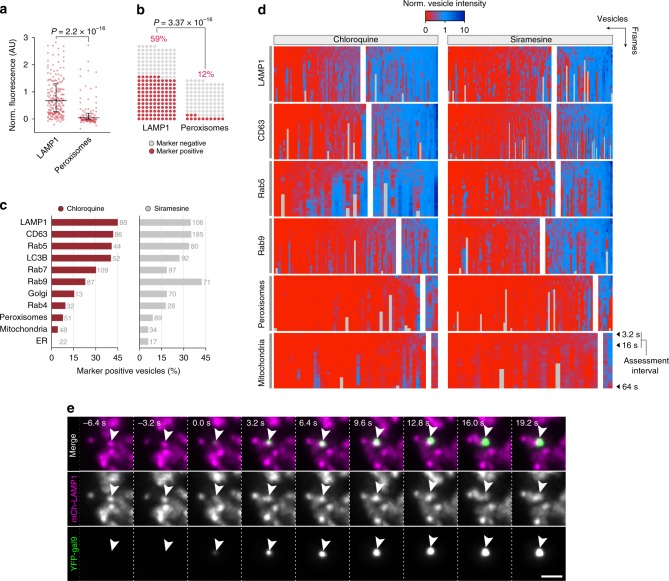
Mapping the compartment identity of disrupted vesicles. HeLa cells expressing YFP–galectin-9 and a mCherry-tagged endosome or organelle marker were treated with membrane disruptive small molecules during widefield live-cell microscopy. De novo induced galectin foci were tracked in 4D, and the marker fluorescence intensity of damaged objects were evaluated between ∼3 and 16 s after detectable galectin recruitment. **a**, **b** Evaluation of galectin-9^+^ vesicles in cells expressing either mCherry-tagged LAMP1 or a peroxisome marker, treated with 1 mM LLOMe. *N* = 195 and 105 vesicles from two independent experiments. **a** Mean intensity of endosomal markers on single vesicles are shown, with median and interquartile range indicated by bars. Two-tailed Mann–Whitney test. **b** Fraction of damaged vesicles classified as positive or negative for the indicated endosomal markers. Two-tailed Fisher’s exact test. **c** Fraction of damaged vesicles with the presence of the indicated endosomal markers. *N* = evaluated vesicles, indicated next to the bars. At least two independent experiments per condition. **d** Heatmaps showing the normalized fluorescence intensity of indicated endosomal and organelle markers on vesicles disrupted by treatment with 60 μM chloroquine or 10 μM siramesine. Single-vesicle traces are organized in columns. Rows represent measurements between ∼3 and 64 s after detectable galectin-9 recruitment, from top to bottom. Traces were classified as negative (left subdivisions) or positive (right subdivisions) for the respective endosomal marker, and sorted by the mean initial trace intensity for visualization. *N* as in **c**. **e** Widefield microscopy images showing galectin-9 recruitment to an endosome labeled with mCherry-LAMP1, indicated by the arrows. Images are representative of four independent experiments. Scale bar, 2 μm. Source data for **a**–**d** are presented in a Source Data file.

To evaluate our method, we first investigated the known lysosomal membrane-permeabilizing agent l-leucyl-l-leucine methyl ester (LLOMe). Intracellularly, LLOMe rapidly accumulates in acidic compartments like lysosomes, where the lysosomal hydrolase cathepsin C mediates its polymerization into highly membranolytic condensation products^35–37^. During treatment with 1 mM LLOMe, the majority of damaged vesicles were indeed LAMP1^+^ (Fig. 6a and Supplementary Fig. 4a). As a negative reference, we used cells expressing mCherry-tagged peroxisomal targeting sequence 1 (PTS1). Since peroxisomes are deficient of cathepsin C, we speculated that this compartment was unlikely to be targeted by LLOMe. In agreement with this, galectin-9 recruitment to PTS1^+^ vesicles was rare. By observer-independent classification, ∼60% of LLOMe-damaged vesicles were LAMP1^+^ within ∼20 s from detectable galectin recruitment (see Methods for details), compared to ∼10% of vesicles with PTS1 (Fig. 6b). To determine the false-positive rate, we visually inspected events were vesicles were PTS1^+^. Some damage events (6 out of 12) showed galectin recruitment to marker-labeled vesicles, with concurrent motion of the structures, suggesting true-positive events. In the remaining cases, galectin-9 was recruited to the vicinity of peroxisomes, implying a false-positive rate around 6%. Hence, high-speed live-cell imaging can determine the compartment identity of newly damaged vesicular structures with high sensitivity and specificity.

We next evaluated a number of endosomal markers in the same way as described above, using chloroquine or siramesine treatment to induce vesicle damage. With chloroquine, ∼45% of damaged vesicles were associated with LAMP1, compared to ∼30% for siramesine (Fig. 6c–e and Supplementary Fig. 4b). For both drugs, similar fractions (∼30%) of the damaged vesicles were positive for the late endosomal markers CD63 and Rab7 (Supplementary Fig. 4c). In contrast, siramesine more often caused damages to Rab9^+^ structures compared to chloroquine (42% and 23%, respectively) (Supplementary Fig. 4d). Rab9 is known to assemble on late endosomes to mediate transport to the *trans*-Golgi network^38^. The fraction of mitochondria and endoplasmic reticulum-positive events with chloroquine or siramesine treatment was around 5%, which is within the false-positive detection rate. This is consistent with the reported absence of galectin-binding glycans on the luminal membrane leaflet of these compartments, making galectins unresponsive to their potential disruption. In summary, markers of several later endosomal compartments are present on 30–45% of the structures damaged by the evaluated small molecules.

**Fig. 7:**
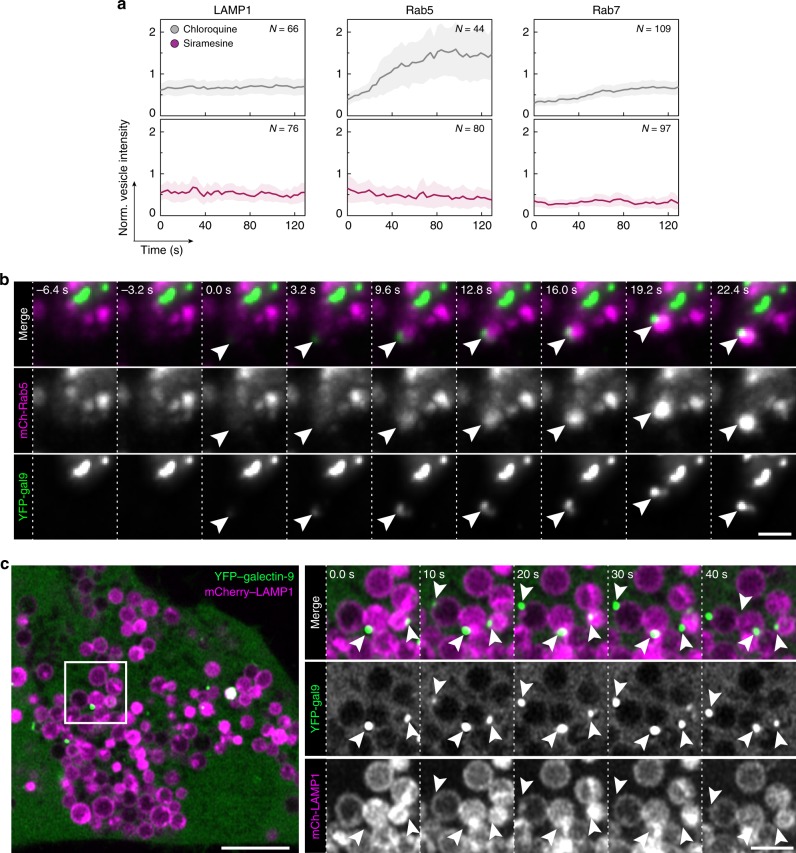
Galectin-9 assembles on LAMP1^+^ vesicles during small-molecule treatment. HeLa cells expressing YFP–galectin-9 and the indicated mCherry-tagged endosome markers were treated with chloroquine or siramesine during live-cell microscopy. **a** The intensity of the indicated endosomal markers measured on vesicles after galectin recruitment. Line is mean, shade is 95% CI. Traces are aligned so that galectin recruitment is first detected at *t* = 0. *N* = vesicles, as indicated in the figure, from at least two independent experiments. **b** Widefield microscopy images showing Rab5 recruitment to an endosome shortly after detectable galectin-9 recruitment, indicated by the arrows. Images are representative of two independent experiments. Scale bar, 2 μm. **c** Live-cell Airyscan confocal microscopy images of HeLa cells expressing mCherry-LAMP1 during treatment with 100 μM chloroquine. Arrows indicate galectin-9 foci on LAMP1^+^ vesicles. Images are representative of two independent experiments. Scale bar, 5 μm; detail, 2 μm. Source data for **a** is presented in a Source Data file.

Interestingly, Rab5, a marker of the early stages of the endosomal system, was present on around one-third of vesicles damaged by both drugs. In addition, to our surprise, damage events triggered by chloroquine frequently induced rapid recruitment of Rab5 to the galectin-marked vesicles (∼43%, *N* = 102 events) (Fig. 7a, b and Supplementary Movie 4). Recruitment was visible typically within ∼30 s after galectin detection, and was often transient, diminishing and even disappearing within only a few minutes. Strikingly, Rab5 was not recruited to vesicles damaged by siramesine. Rab7 was also exclusively recruited to vesicles damaged by chloroquine, but not as pronounced as Rab5. The clear association between galectin response, indicating vesicle rupture, and Rab5 recruitment suggests that Rab5 is involved in the immediate response to endolysosomal membrane damage. Indeed, Rab5 was recently reported to respond to mitochondrial membrane damage^39,40^. During the total observation period of ∼2 min, additional recruitment of the autophagosome protein LC3B or LAMP1 to the damaged vesicles was not seen.

Using high-resolution Airyscan live-cell confocal microscopy, galectin-9 could be clearly visualized as discrete foci on enlarge LAMP1^+^ vesicles during chloroquine treatment (Fig. 7c and Supplementary Fig. 5a). Thus, in addition to intra-luminal decoration of damaged vesicles with galectin (Supplementary Fig. 5b), galectin-9 can also form aggregates limited to one pole of the vesicle membrane. In all, our data suggest that different subsets of endolysosomal compartments are targeted by these small molecules, consistent with the lower hit-rate of vesicles containing dextran or chol-siRNA using siramesine, and its more modest knockdown enhancement.

### Drug-induced chol-siRNA release is cell type dependent

A key parameter determining the applicability of a specific small-molecule release enhancer is the relationship between the number of galectin foci per cell and the toxicity. In a recent small-molecule high-throughput screen, the μ-opioid receptor agonist loperamide was identified to cause high numbers of galectin-3 foci in MCF7 breast cancer cells, with minimal toxicity^41^. Given these observations, we decided to evaluate the effect of loperamide on chol-siRNA delivery.

**Fig. 8:**
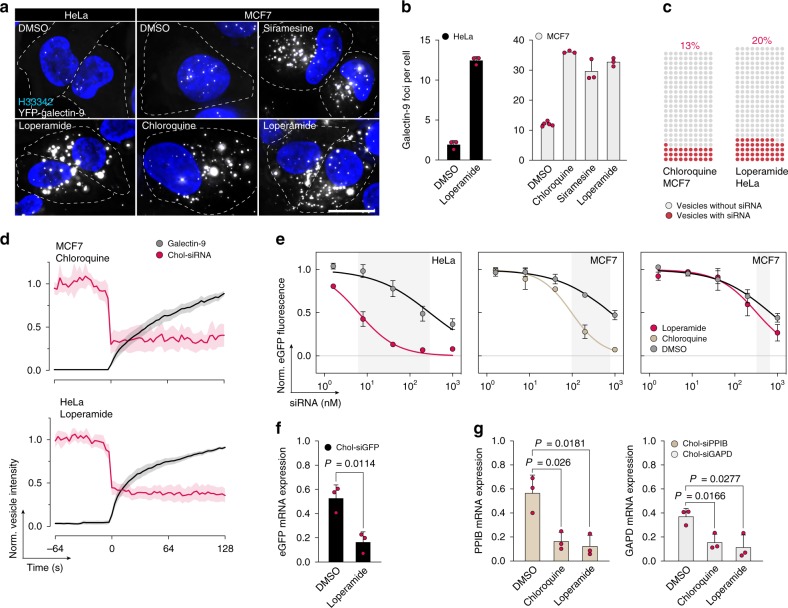
Small-molecule-induced cholesterol-siRNA release is cell type dependent. HeLa or MCF7 cells expressing YFP–galectin-9 were treated with 10 μM siramesine, 100 μM chloroquine, or 20 μM loperamide for 24 h, and imaged with widefield microscopy. **a** Representative images from three independent experiments. Scale bar, 20 μm. **b** Number of galectin-9 foci per cell after drug treatment. Mean ± s.d. from three independent experiments. **c**, **d** HeLa or MCF7 cells expressing YFP–galectin-9 were incubated with 200 nM DY547-chol-siRNA for 6 h, and treated with 25 μM loperamide or 60 μM chloroquine during live-cell widefield microscopy. **c** Fraction of damaged endosomes containing chol-siRNA before galectin-9 recruitment. *N* = 230 and 240 vesicles from two independent experiments, respectively. **d** Quantification of chol-siRNA release by single-vesicle tracking. Line is mean, shade is 95% CI. Traces are aligned so that galectin recruitment is first detected at *t* = 0. *N* = 19 and 35 vesicles from two independent experiments. **e** HeLa-d1-eGFP or MCF7-d1-eGFP cells were incubated with chol-siGFP as indicated for 6 h, followed by treatment with DMSO as control, 25 μM loperamide, or 100 μM chloroquine for 18 h. eGFP knockdown was determined by flow cytometry. Mean ± s.d. is shown. *N* = 4, 3, and 3 independent experiments. **f** HeLa-d1-eGFP or **g** HeLa wild-type cells were incubated with 40 nM chol-siGFP, 200 nM chol-siPPIB, or chol-siGAPD for 6 h, followed by treatment with 20 μM loperamide or 60 μM chloroquine for 18 h. Knockdown was determined by real-time quantitative PCR. Mean ± s.d. from three independent experiments. Two-tailed Student’s *t* test. Source data for **b**–**g** are presented in the Source Data file.

Loperamide treatment triggered the formation of numerous galectin-9 foci, in both HeLa and MCF7 cells (Fig. 8a, b). We also confirmed that siramesine and chloroquine were likewise able to trigger galectin-9 foci in MCF7 cells. Interestingly, intranuclear galectin-9 foci were more numerous in MCF7 than HeLa cells. Live-cell microscopy revealed that the fraction of damaged vesicles containing siRNA was ∼20% with loperamide in HeLa cells (Fig. 8c). The release efficiency and kinetics of individual release events with loperamide or chloroquine treatment, in HeLa and MCF7, respectively, was similar to what was seen in HeLa cells using chloroquine or siramesine (Fig. 8d). As suggested by the increased rate of siRNA release events, d1-eGFP knockdown was substantially improved by loperamide treatment in HeLa cells, with a ∼47-fold IC_50_ reduction, enhancing knockdown to ∼90% with 20 nM siRNA (Fig. 8e). In comparison, without a small-molecule enhancer, knockdown in control cells was <60% even with 1000 nM siRNA. The enhancement was comparable with that of chloroquine for concentrations with similar cell viability (Supplementary Fig. 6a). In contrast, loperamide had only a small effect on d1-eGFP knockdown in MCF7 cells, while chloroquine improved knockdown IC_50_ ∼8-fold (Fig. 8e). Improved knockdown of d1-eGFP with loperamide was also confirmed at the mRNA level (Fig. 8f).

Chol-siRNA uptake in MCF7 cells was approximately half that of HeLa (Supplementary Fig. 6b). In addition, MCF7 cells are substantially larger, possibly requiring more siRNA molecules for an equivalent effect. The lower knockdown enhancement (and general knockdown efficiency) seen in MCF7 is thus a combined effect of lower uptake, larger cell size, and importantly, a low rate of damages to vesicles containing siRNA—in particular with loperamide treatment.

To verify that the drug-induced knockdown improvement was not isolated to the *eGFP* gene, we also used cholesterol-conjugated siRNAs targeting two other genes: glyceraldehyde-3-phosphate dehydrogenase (*GAPDH*) and peptidyl-prolyl isomerase B (*PPIB*, also known as cyclophilin B). Target mRNA knockdown was similarly improved for both of these genes with chloroquine or loperamide treatment in HeLa cells (Fig. 8g). This further demonstrates that the application of small-molecule substances to induce endosomal damages, producing meaningful knockdown enhancement, is not limited to a single-cell type or substance. However, our data indicate that the effects of such small-molecule drugs can be cell type specific.

### Induced siRNA release improves knockdown in tumor spheroids

**Fig. 9:**
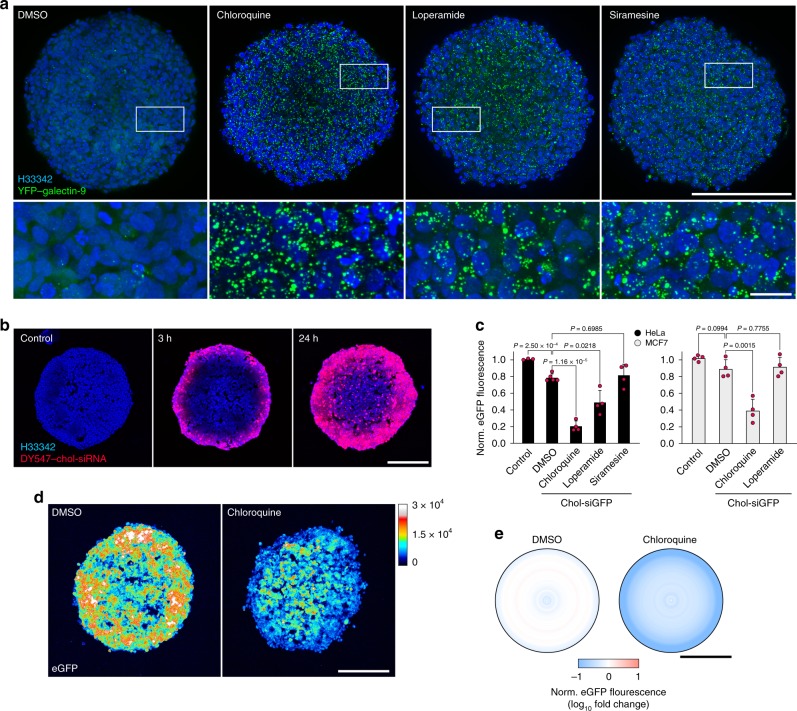
Induced endosomal damages improves chol-siRNA-mediated knockdown in tumor cell spheroids. **a** HeLa-galectin-9-YFP cell spheroids incubated with 100 μM chloroquine, 20 μM loperamide, or 10 μM siramesine for 20 h imaged with confocal microscopy after optical clearing. Maximum intensity projections of 45-μm *z*-stacks are shown. Images are representative of three independent experiments with at least three spheroids per condition. **b** Cryosections of HeLa cell spheroids incubated with 1 μM DY547-chol-siRNA for 3 or 24 h, imaged with confocal microscopy. Maximum intensity projections of 15-μm *z*-stacks are shown. Images are representative of at least three spheroids per condition, from three independent experiments. **c** HeLa-d1-eGFP or MCF7-d1-eGFP cell spheroids were incubated with 1 μM chol-siGFP and the indicated small molecules for 3 h, followed by 27 h incubation with small-molecule-supplemented medium only. eGFP knockdown was determined by flow cytometry after spheroid dissociation. Mean ± s.d. is shown. *N* = 3 (HeLa control), 5 (HeLa DMSO + siGFP) or 4 independent experiments. Two-tailed Student’s *t* test. **d** HeLa-d1-eGFP cell spheroids were treated as in **c**, cryosectioned and imaged using confocal microscopy. Images are mean intensity projections of 5-μm *z*-stacks, representative of three independent experiments with at least four spheroids per condition. **e** Quantification of spheroid eGFP fluorescence intensity in **d**, measured in concentric layers from spheroid margins, and normalized to control-treated spheroids. Bar shows mean scale of all spheroids. All scale bars are 200 μm; details 20 μm. Source data for **c**, **e** are presented in the Source Data file.

We next assessed the possibility of detecting induced vesicle damage in cancer cell spheroids established from HeLa or MCF7 cells expressing YFP–galectin-9. Few galectin-9 foci were detectable in tumor spheroids without small-molecule treatment, whereas all tested compounds induced formation of abundant galectin-9 foci (Fig. 9a, Supplementary Fig. 7a, and Supplementary Movie 5). Galectin-9 foci were detectable also in the more central regions of the spheroids. This was confirmed by cryosectioning and subsequent imaging of the core using confocal microscopy (Supplementary Fig. 7b). When spheroids were incubated with chol-siRNA for up to 24 h, the siRNA penetrated through the outer cell layers within a few hours, successively distributing toward the core region (Fig. 9b).

To test if treatment with any of the membrane-disrupting small molecules could improve knockdown in tumor cell spheroids, we incubated HeLa-d1-eGFP or MCF7-d1-eGFP spheroids with 1 μM chol-siGFP and the indicated small molecule for 3 h, followed by 27 h small-molecule treatment only. After spheroid dissociation, d1-eGFP expression was evaluated using flow cytometry. In HeLa cell spheroids, knockdown was improved from 21% to 79% with chloroquine treatment, and to 51% with loperamide (Fig. 9c). No improvement was observed with siramesine treatment. In MCF7 spheroids, chloroquine improved knockdown ∼6-fold (from 11% to 61%), while loperamide had no effect. Thus, the drug-induced knockdown enhancement in tumor spheroids are consistent with the effects observed in 2D cell cultures.

Next, we evaluated the spatial distribution of the knockdown enhancement within the tumor spheroid. Central cryosections of HeLa-d1-eGFP spheroids treated with chol-siGFP, with or without chloroquine, were imaged by confocal microscopy (Fig. 9d). Quantification of the d1-eGFP fluorescence intensity in concentric layers of the spheroid revealed that the effect of chloroquine was most pronounced in the outer layers, but knockdown enhancement was observed throughout the spheroid (Fig. 9e). Thus, chloroquine can improve cholesterol-siRNA delivery throughout tumor spheroids of at least 400–500 μm in diameter.

## Discussion

To improve the delivery of macromolecular oligonucleotide drugs—which is critical for their potential use in more clinical settings—a better understanding of the endosomal escape bottleneck is required. In this work, we demonstrate the applicability of galectin-9 as a highly sensitive membrane damage sensor, to directly visualize and measure properties of endosomal escape of ligand-conjugated siRNA. We used this approach to characterize drug-induced endosomal escape of chol-siRNA, facilitated by small molecules with membrane disruptive properties. We show that these compounds improve delivery of chol-siRNA, enhancing target knockdown up to ∼47-fold. Moreover, our data reveal that vesicle-disrupting drugs target and damage a diverse set of endolysosomal compartments, ultimately reflected in their ability to potentiate knockdown with varying success in vitro and in tumor cell spheroids.

Other studies have investigated the use of small molecules for enhancing knockdown mediated by chol-siRNA and LNP-siRNA^19–22^. In these studies, the identified small molecules acted through either facilitating uptake or by effects on intracellular processes—potentially including endosomal escape. However, without direct observation of membrane damage response nor siRNA release, statements about endosome disruption as the basis of improved knockdown by such small molecules have been largely inferential.

In contrast, directly visualizing endosomal escape of ligand-conjugated siRNA enabled us to determine the productive pathway of delivery, and to measure properties of the release process that until now have been completely unknown. These parameters include the release frequency, kinetics, and efficiency of siRNA escape. Additionally, since we evaluate individual productive or non-productive damage events—and not averages or ensembles—our data capture the spread of these parameters. Thus, we are able to estimate the key properties of intracellular siRNA delivery, where optimization could shift a delivery strategy toward higher efficiency. This concept can conceivably be adapted to the investigation of other delivery-enhancing methods, including vesicle-disrupting peptides or polymers^17,18,42^.

The sensitivity and rapid kinetics of the galectin-9-based assay presented here allowed us to characterize the compartments damaged by small-molecule drugs in unprecedented detail. By quantitative analysis of ∼2000 damage events, we show that both chloroquine and siramesine damage a diverse set of vesicles along the endosomal system, with an emphasis on later endosomal or lysosomal structures. A key observation was the marked difference in the probability of vesicles containing chol-siRNA being damaged by the evaluated small molecules. Indeed, we demonstrate that this is a critical factor explaining the substantially higher knockdown enhancement observed with chloroquine compared to siramesine. Analysis of this “hit-rate” will arguably be key to optimize diverse delivery strategies.

Previous work on LNP-mediated delivery showed that siRNA is released during endosomal maturation, from vesicles marked by Rab5 and to a lesser extent Rab7^9^. Interestingly, no release was seen from LAMP1^+^ structures, indicating that accumulation of LNPs in lysosomes is a non-productive end point. Using the small molecules evaluated here, we found that vesicles releasing chol-siRNA were consistently LAMP1^+^. Investigating endosomal escape of ligand-conjugated siRNA is a significantly harder proposition than the characterization of compartments releasing LNP-formulated siRNA, as the particulate payload vastly facilitates tracking of the disrupted structure—both before and after the damage event. Whereas release from LNPs only occurs during a narrow ∼10 min window of opportunity during endosomal maturation, ligand-conjugated siRNA can escape from late endosomes and lysosomes to successfully mediate RNAi. In this way, these compartments could potentially act as reservoirs for multiple rounds of adjuvant-induced siRNA release, as has been proposed for polymer-formulated siRNA^22^. However, whether galectin-9 is as efficient an endosomal release sensor using other delivery strategies remains to be seen.

We demonstrate improved target knockdown in tumor cell spheroids using two cancer cell lines, consistent with the distribution of chol-siRNA and induced galectin-9 foci throughout the spheroids. It remains to be seen how our findings would translate to the in vivo setting. When injected intratumorally in a glioblastoma model, modified cholesterol-siRNA had a wide distribution throughout the tumor, producing 45–77% knockdown of a human target gene^16,43^. Localized administration of a small-molecule adjuvant could potentiate knockdown and reduce the required dosing, limiting adverse neurotoxic effects from high intracerebral concentrations of hydrophobically modified siRNA^43^. Additionally, tumor cells have been proposed to be more sensitive to small-molecule-induced membrane damage than other tissues^24^.

To conclude, we present a strategy to detect and characterize endosomal escape of ligand-conjugated siRNAs. We show that membrane-destabilizing small molecules can act as enhancers of siRNA knockdown through induction of endosomal escape—a fundamental efficiency-limiting hurdle in the delivery of siRNA therapeutics. Going forward, we believe this work will contribute to illuminate the mechanisms mediating successful endosomal escape of nucleic acid therapeutics and how it can be improved, ultimately advancing this new therapeutic modality to broader clinical use.

## Methods

### Cell culture and reagents

HeLa and MCF7 cells were purchased from the American Type Culture Collection, and were confirmed to be free from mycoplasma. Cells were cultivated in Dulbecco’s modified Eagle’s medium (DMEM) (Sigma-Aldrich, St. Louis, MO, USA) supplemented with 10% fetal bovine serum (FBS, Gibco), 100 U mL^−1^ penicillin and 100 mg mL^−1^ streptomycin (Gibco) and 2 mM glutamine (Thermo Fisher Scientific, Waltham, MA USA) at 37 °C and 5% CO_2_. Imaging medium was used for live-cell microscopy experiments, consisting of FluoroBrite DMEM (Gibco), supplemented with 10% FBS, 2 mM glutamine, and 2 mM HEPES. For microscopy experiments, 5 × 10^4^ cells were plated per well in 8-well Lab-Tek II chambered cover glass slides (Nunc, Rochester, NY, USA) one day before experiments. For galectin cross-evaluation, plasmid transfections were performed using Lipofectamine 2000 (Invitrogen, Thermo Fisher Scientific). For all other experiments, a Neon Transfection System (Thermo Fisher) was used. Cells were seeded in 6-well plates at 3 × 10^5^ cells per well one day before Lipofectamine 2000 transfection. Transfections were carried out overnight at ∼70% cell confluency, according to the manufacturer’s protocol. For Neon transfection, 5 × 10^5^ cells were used with the 100-μL tip size and cell-type-specific protocol provided by the manufacturer. Transfected cells were used in experiments between 24 and 48 h after transfection.

Siramesine fumarate salt, amitriptyline hydrochloride, chloroquine diphosphate salt, loperamide hydrochloride, LLOMe, dimethyl sulfoxide (DMSO), bovine serum albumin (BSA), and Triton X-100 were all from Sigma. Alexa Fluor 647-labeled 10 kDa dextran was from Thermo Fisher. Drug-containing medium were prepared fresh from stock in fully supplemented DMEM or imaging medium for all experiments.

Plasmids encoding mCherry-Rab9a were a gift from Yihong Ye (Addgene #78592). Plasmids encoding mCherry-LC3B were a gift from David Rubinsztein (Addgene #40827). Plasmids encoding mCherry-tagged calnexin, TGN46, LAMP1, peroxisomes, Golgi, Mito, Rab4a, Rab5a, and Rab7a were a gift from Michael Davidson (Addgene #55005, #55145, #55073, #55073, #55052, #55102, #55125, #55126, #55127). Plasmid encoding mCherry-tagged CD63 was a gift from M. Belting. Plasmid encoding N-terminal eGFP-tagged human LAMP1 was a gift from J. Lieberman. Plasmids encoding YFP-tagged galectins were gifts of F. Randow. Plasmid encoding mCherry-tagged galectin-3 was a gift from H. Leffler.

Rat anti-galectin-3 antibody (M3/38)^44^ was produced in-house in the lab of H. Leffler. Cy3-conjugated AffiniPure donkey anti-rat immunoglobulin G (IgG) secondary antibodies were from Jackson ImmunoResearch Laboratories Inc. (West Grove, PA, USA).

Chol-conjugated siRNAs (Accell) were from Dharmacon (Lafayette, CO, USA), with the following target sequences: non-targeting #1: UGGUUUACAUGUCGACUAA; eGFP: GCCACAACGUCUAUAUCAU; cyclophilin B: GCCUUAGCUACAGGAGAGA; GAPD: UGUGAACCAUGAGAAGUA; Red non-targeting: UGGUUUACAUGUCGACUAA. Custom-synthesized cholesterol-conjugated Alexa Fluor 647-labeled siRNA targeting eGFP: sense, 5′-GGCUACGUCCAGGAGCGCAtst-AF647-3′; antisense, 5′-UGCGCUCCUGGACGUAGCCtst-CholTEG-3′, where lowercase denotes a deoxynucleotide and “s” indicates a phosphorothioate linkage.

The following PCR primers were used: eGFP forward, 5′-ACGTAAACGGCCACAAGTTC-3′ and eGFP reverse, 5′-AAGTCGTGCTGCTTCATGTG-3′ (Sigma); GAPDH forward, 5′-CTGGGCTACACTGAGCACC-3′ and GAPDH reverse, 5′-AAGTGGTCGTTGAGGGCAATG-3′ (Invitrogen); ACTB forward, 5′-AGCACAGAGCCTCGCCTTT-3′ and ACTB reverse, 5′-GGAATCCTTCTGACCCATGC-3′; PPIB forward, 5′-TCTGTCTTGGTGCTCTCCACCT-3′; PPIB reverse, 5′-AACGCAGGCAAAGACACCAACG-3′ (Integrated DNA Technologies, Leuven, Belgium).

### Immunofluorescence

At the end of the experiment, cells were washed with phosphate-buffered saline (PBS) and fixed with 4% paraformaldehyde (PFA) for 10 min at room temperature (RT). Cells were washed twice with PBS, permeabilized with 0.1% Triton X-100 for 10 min, washed twice with PBS, and incubated with 2% BSA in PBS for 30 min. Cells were then incubated with anti-galectin-3 IgG diluted 1:500 in 2% BSA PBS for 1 h at RT. Cells were washed three times with PBS, and 1:1000 secondary anti-rat IgG and 100 ng mL^−1^ Hoechst 33342 prepared in 2% BSA PBS was added for 1 h. Cells were washed three times with PBS, and the PBS was added to the wells. Images were acquired with a confocal microscope.

### Galectin recruitment evaluation

HeLa cells transiently expressing mCherry–galectin-3 and YFP-labeled galectin-1, -3, -8, or -9 were treated with 10 μM siramesine while images were acquired using a confocal microscope. Galectin recruitment events were manually identified and tracked in maximum intensity projections of *z*-stacks using the Manual Tracking plugin in Icy version 1.6.1.1. A four-pixel diameter object mask was used. Pre-event measurements were confined to the exact location of the subsequent event, whereas post-event tracking followed the intensity maximum of galectin foci. The time of the first frame with visually detectable galectin recruitment was set to *t*_0_ = 0. Image background intensity was subtracted from the measured values, and traces were normalized to the mean pre-event galectin signal ∼10–90 s before *t*_0_. Galectin fold recruitment was calculated as the mean intensity of foci ∼90–105 s after *t*_0_, divided by the mean cell background measurement ∼10–25 s before *t*_0_.

### Cytotoxicity assay

Cells were plated in 48-well plates, 2.5 × 10^4^ cells per well. The following day, the medium was replaced with fully supplemented DMEM, and drugs were diluted in sterile filtered deionized water and added to the medium to yield the final concentrations. After 24 h, the medium was removed and transferred to 5 mL 75 × 12 mm polystyrene FACS tubes. Cells were washed once with PBS, and the PBS was transferred to the corresponding tube. After dissociating cells with trypsin, DMEM was added and cell suspensions were transferred to corresponding FACS tubes. Samples were centrifuged at 300 × *g* for 5 min and the supernatant was decanted. Cell pellets were resuspended in 2.5 μM propidium iodide in PBS prepared from stock, and analyzed using flow cytometry. Viable and apoptotic populations were gated in FL3/FSC plots, and cell viability was calculated as the number of viable cells divided by all gated cells. Means of duplicate samples were calculated for each experiment.

### Chol-siRNA internalization

For time-lapse microscopy of chol-siRNA internalization, OptiMEM supplemented with 10 ng mL^−1^ Hoechst 33342 was added to the cells before transferring the sample to the microscope incubator. Immediately before starting image acquisition, DY547-labeled chol-siRNA prepared in OptiMEM was added to yield a final concentration of 100 nM. Controls only received OptiMEM. For each experiment, eight positions in the chol-siRNA-containing well and four positions in the control well were monitored for 24 h, acquiring five *z*-planes per field of view with a confocal microscope. CellProfiler was used to detect cell nuclei, cell boundaries and galectin-9 foci, and images with segmented and labeled cells were exported as tiff-files. A MATLAB script was then used for image analysis by importing raw tiff-images and segmented objects. Individual cells were masked in the chol-siRNA channel using labeled cell objects. Image background intensity was calculated as the median pixel from the *z*-plane with the lowest intensity value after masking all cells. Individual cells were masked with a 5-pixel additional eroded margin, to exclude chol-siRNA present on the plasma membrane. The mean value of the remaining pixels from the *z*-plane with highest intensity was used as a measure of the intracellular siRNA quantity, after subtraction of the image background intensity.

For analysis of lysosomal localization of chol-siRNA, cells expressing GFP-LAMP1 were incubated with 200 nM DY547-labeld chol-siRNA for 6 h. Cells were either pre-treated with chloroquine 6 h before siRNA addition, received chloroquine together with siRNA, or after 6 h siRNA incubation. Cells were fixed with 4% PFA for 10 min at RT, at the end of the siRNA incubation, or after 6 h additional incubation in complete DMEM with chloroquine or DMSO. Cells were washed three times with PBS and incubated in PBS supplemented with 100 ng mL^−1^ Hoechst 33342. Single *z*-plane images were acquired using an Airyscan confocal microscope in super-resolution mode. Cell boundaries and image background regions were manually identified in Fiji. CellProfiler was used to detect LAMP1-labeled objects. A MATLAB script was then used for image analysis by importing raw tiff-images and segmented objects. After background intensity subtraction, the lysosomal to total chol-siRNA ratio was calculated as the siRNA intensity sum of GFP-LAMP1-labeled objects divided by the sum of the siRNA intensity of the entire cell.

For comparison of chol-siRNA internalization in HeLa and MCF7 cells, cells were incubated with 200 nM DY547-labeled chol-siRNA for 6 h, fixed with 4% PFA for 10 min at RT, washed three times with PBS, and incubated in PBS supplemented with 100 ng mL^−1^ Hoechst 33342. Single *z*-plane images were acquired using a confocal microscope. CellProfiler was used to detect cell boundaries and measure the mean siRNA fluorescence intensity per cell.

### Drug-enhanced eGFP knockdown

HeLa-d1-eGFP or MCF7-d1-eGFP cells were plated in 48-well plates, 3 × 10^4^ cells per well. Unless otherwise stated, incubations with chol-siGFP were performed in OptiMEM for 6 h, followed by drug treatment for 18 h in fully supplemented DMEM. At the end of the experiment,  cells were washed with PBS and dissociated by trypsin treatment. Fully supplemented DMEM was added and the suspensions were transferred to a 96-well V-bottomed microplate and centrifuged at 400 × *g* for 5 min. The supernatant was decanted and cells were resuspended in PBS, followed by centrifugation again as stated. The supernatant was decanted, and the cells were resuspended in 1% BSA PBS for direct analysis using flow cytometry. For experiments with multiple read-out time points, cells were resuspended in 4% PFA on ice for 20 min, followed by centrifugation as stated, resuspension in PBS, centrifugation, and resuspension in 1% BSA PBS. Samples were kept at 4 °C in dark until flow cytometry analysis at the end of the experiment.

### Galectin foci and cytosolic dextran quantification

For drug-induced endosomal damage quantification, HeLa or MCF7 cells stably expressing YFP–galectin-9 cells were treated with drugs prepared in complete DMEM at the concentrations indicated for 24 h. The medium was removed and cells were washed once with PBS. For HeLa cells, imaging medium supplemented with 100 ng mL^−1^ Hoechst 33342 was added, cells were transferred to a heated microscopy incubation chamber, and images were acquired using a confocal or widefield microscope. MCF7 cells were fixed with 4% PFA for 10 min at RT, washed three times with PBS, and incubated with PBS containing 100 ng mL^−1^ Hoechst 33342 overnight and imaged with a widefield microscope. CellProfiler was used to detect cell nuclei, cell boundaries, and galectin-9 foci in maximum intensity projection images.

For continuous monitoring of galectin-9 response during 24 h drug treatment, cells were washed once with PBS, and imaging medium supplemented with 20 ng mL^−1^ Hoechst 33342 was added before transferring the cells to a heated microscope incubator. Cells were monitored in four microscopy slide wells, three positions per well, with the conditions indicated using a confocal microscope. Imaging positions were selected manually before adding 10% of the final volume supplemented with siramesine or chloroquine to yield the concentrations indicated. Image acquisition was then initiated immediately, acquiring 10 *z*-plane image stacks per position with 15 min interval for 24 h. Images were analyzed with CellProfiler as described above.

For quantification of cytosolic dextran after drug treatment, cells were incubated with 200 μg mL^−1^ Alexa Fluor 647-labeled dextran prepared in OptiMEM for 4 h, washed three times with PBS, and incubated in complete DMEM for 2 h. Cells were then washed with PBS and imaging medium supplemented with 100 ng mL^−1^ Hoechst 33342 was added. Cells were immediately transferred to a heated microscope incubator and images were acquired with a confocal microscope before adding the drugs at the concentrations indicated. Cells were transferred to an ordinary cell culture incubator in between imaging time points. Images were acquired again after 8 and 16 h drug treatment. An Airyscan detector was used in confocal mode for imaging of dextran. A single *z*-plane was acquired in the dextran channel, followed by five *z*-planes in the remaining channels to properly capture the galectin foci. For image segmentation, the dextran and galectin channels were merged using a MATLAB script to enhance the contrast between cell outlines and image background. Segmentation of cells and galectin foci was then performed with CellProfiler. Images containing segmented and labeled cells or galectin foci were exported as tiff-files. A MATLAB script was then used to read raw tiff-images and segmented objects to perform image analysis. Individual cells were first masked in the dextran channel using labeled cell objects. Dextran-containing endosomes were identified as having pixel values above a prespecified threshold and were subsequently masked, together with a 5-pixel additional dilated margin. The median intensity value of the remaining pixels was used as a measure of the cytosolic dextran. The mean cytosolic intensity of all measured control cells was subtracted from the values of individual cells in the drug-treated samples at the corresponding time point, to correct for the contribution of out-of-focus stray light from dextran-containing objects. The number of galectin-9 foci per cell was then determined using the labeled objects images segmented in CellProfiler.

### Single-vesicle endosomal release

HeLa or MCF7 cells stably expressing YFP–galectin-9 were plated in microscopy slides as stated. The next day, the medium was removed and cells were washed once with PBS. For dextran release, cells were incubated with 200 μg mL^−1^ Alexa Fluor 647-labeled dextran prepared in OptiMEM for 4 h, washed three times with PBS, followed by 2 h chase in complete DMEM. For siRNA release, cells were incubated with 200 nM DY547-labeled chol-siRNA prepared in OptiMEM for 6 h. At end of incubations, the medium was removed, cells were washed once with PBS, and imaging medium supplemented with siramesine, chloroquine, or loperamide was added to the cells. Typically, microscopy slides were transferred to a heated microscope incubator after 1–3 h treatment. Images were then acquired using widefield microscopy and processed as stated below.

For tracking of siRNA- or dextran-containing vesicles before galectin recruitment was detected, release events were identified and tracked in Fiji using the Manual Tracking plugin. Tracking coordinates were exported and used with an in-house developed MATLAB program for image quantification as follows. An object mask was fitted to the tracked vesicle, and the mean object intensity was estimated as the maximum single-plane fitted measurement in the *z*-stack. After galectin recruitment, automated tracking of the galectin foci was performed as described below. Traces were aligned in time so that *t*_0_ = 0 corresponds to the first frame with detectable galectin recruitment. Local fluorescence background was estimated as the median pixel intensity in a circular area ∼0.8 μm (8 pixels) outside the object mask. A local background rolling average was calculated over 11 frames and subtracted from the vesicle intensity measurement. Vesicle siRNA or dextran traces were normalized to the mean vesicle intensity ∼3–60 s before detectable galectin recruitment. Galectin traces were normalized to the maximum value.

### Mapping of damaged endosomal compartments

HeLa cells stably expressing YFP–galectin-9 and transient mCherry-tagged endosomal or organelle markers were prepared as described above. Cells were treated with 10 μM siramesine, 60 μM chloroquine, or 1 mM LLOMe while acquiring images with a widefield microscope. Object detection and tracking was performed as described below. Traces were aligned in time so that *t*_0_ = 0 corresponds to the first frame with detectable galectin recruitment. Local fluorescence background was estimated as the median pixel intensity in a circular area ∼0.8 μm (8 pixels) outside the object mask. A local background rolling average was calculated over 11 frames and subtracted from the vesicle intensity measurement. Vesicle traces were normalized to the mean intensity of all marker-labeled objects in the corresponding cell, calculated as follows. First, log_10_-transformed cell pixel intensity values above the mean + 1 × s.d. were assigned to marker-labeled objects. Second, the nonlogarithmic mean of marker-labeled objects was calculated at the beginning and end of the acquisition, and a linear regression model was fitted and used for time-matched trace normalization. The signal-to-noise ratio (SNR) of marker-labeled objects was estimated as the difference between the mean object intensity and mean image (detector) background, divided by the s.d. of the image background pixel values. Cells having marker-labeled objects with an SNR <50 was excluded from the analysis. The marker intensity of vesicles was assessed in an interval ∼3–16 s after galectin recruitment was detected, corresponding to five measurements. Vesicles with a normalized mean marker intensity above 0.5 during the assessment interval were classified as marker positive. Measurements of marker-positive vesicles showing a ratio between trace s.d. and mean intensity above 1 were considered unreliable, likely due to influence by the presence of positive vesicles in the near vicinity, and were reclassified as marker negative.

For live-cell Airyscan confocal microscopy, a single *z*-plane was acquired in super-resolution mode. Bleach correction was applied for mCherry-LAMP1 using Fiji.

### Tumor cell spheroid formation and galectin-9 assays

Spheroids were formed by plating 5 × 10^3^ HeLa or 1×10^4^ MCF7 cells, stably expressing YFP–galectin-9, per well in a 96-well spheroid microplate (Corning, Kennebunk, ME, USA) in complete DMEM. For the evaluation of galectin-9 foci formation by drug treatment, the medium was removed from the wells after 3 days. Complete DMEM supplemented with 0.1% DMSO, 20 μM loperamide, 10 μM siramesine, or 100 μM chloroquine prepared fresh from stock was added. After 20 h drug treatment, the medium was removed and spheroids were washed once with PBS before fixation with 4% PFA at 4 °C for 20 min in dark. The PFA was removed and spheroids were washed three times with PBS.

Preparation of intact spheroids for imaging was performed by transferring spheroids onto microscope glass slides using a pipette, and aspirating the PBS. Two drops (∼20 μL) Cytovista 3D Cell Culture Clearing Reagent (Thermo Fisher) were applied on each spheroid. After incubating for 2 h at RT in dark, spheroids were aspirated and transferred to 0.5 mL Eppendorf tubes containing PBS to remove residual clearing solution. Spheroids were then aspirated and transferred to individual tubes containing PBS supplemented with 3 μg mL^−1^ Hoechst 33342, and then kept in the dark at 4 °C. For imaging, spheroids and a small volume PBS were transferred onto a No. 1.5 borosilicate cover glass mounted in an Attofluor Cell Chamber (Thermo Fisher).

For cryosectioning, spheroids were placed in 15 × 15 × 5 mm base molds and OCT Cryomount medium (Histolab, Gothenburg, Sweden) was added and molds were placed at −20 °C for at least 2 h.  Approximately 25 μm-thick frozen sections were cut using a Leica CM3050 cryostat, and sections were placed on SuperFrost Plus microscope glass slides (Thermo Fisher). Dako Fluorescence Mounting Medium (Agilent, Santa Clara, CA, USA) supplemented with 5 μg mL^−1^ Hoechst 33342 was applied and a No. 1.5 borosilicate cover glass was placed on top and secured with transparent nail polish.

Spheroids were imaged using a confocal microscope with a water-immersion objective. Typically, *z*-stacks were acquired with 1- or 2-μm intervals, with 45- or 90-μm imaging depth of cleared HeLa or MCF7 spheroids, respectively, or 15-μm imaging depth for cryosections.

### eGFP knockdown in tumor cell spheroids

Two days after spheroid formation, the medium was removed and cells were washed once with OptiMEM. Chol-siGFP or control chol-siRNA was prepared in OptiMEM supplemented with 100 μM chloroquine, 10 μM siramesine, or 20 μM loperamide. Spheroids were incubated with siRNA and drugs for 3 h, washed with PBS once, and incubated with complete DMEM supplemented with drugs for an additional 27 h.

For flow cytometry analysis, the medium was removed, spheroids washed once with PBS, and dissociated with trypsin at 37 °C for 10–15 min with pipetting every 5 min. Cell suspensions were transferred to a 96-well V-bottomed microplate, centrifuged at 400 × *g* for 5 min, resuspended in PBS, centrifuged again, and resuspended in 1% BSA PBS before flow cytometry analysis.

For confocal microscopy, spheroids were prepared and cryosectioned as described above. Spheroids were imaged using a ×63 1.40 NA objective lens, acquiring five *z*-planes with 2-μm interval. Spheroid region of interest (ROI) masks and mean intensity projections of image stacks were created using Fiji. A MATLAB script was then used to quantify eGFP fluorescence intensity of individual spheroids. In brief, images were scaled so that spheroids had a radius of ∼200 pixels, using the nearest-neighbor interpolation method. The image background upper limit was calculated as the mean + 3 × s.d. of the image background after masking of the spheroid. Spheroid pixels below this value were set to NaN and omitted from the analysis. The mean eGFP fluorescence intensity was then calculated for shells with 1-pixel thickness, from the margin towards the center of the spheroid. Mean values were mapped on the shells of a circle with a 200-pixel radius, corresponding in location with the spheroid shells. Mapped values from chol-siGFP treated spheroids were corrected for cell background fluorescence calculated in HeLa wild-type spheroids, and normalized to HeLa-d1-eGFP spheroids treated with control chol-siRNA with the addition of DMSO or chloroquine. For data visualization, mapped values (ratios) were multiplied by 10^4^, log_10_ transformed, multiplied by 10^3^, and exported as uint16 tiff-images. Look-up tables were assigned in Fiji with brightness scaled from 3–5 × 10^3^.

### Flow cytometry

For cell viability evaluation, samples were analyzed on an Accuri C6 Flow Cytometer (Becton Dickinson, Franklin Lakes, NJ, USA). For eGFP knockdown analysis, a FACSAria IIu Flow Cytometer (Becton Dickinson) was used, operating under BD FACSDiva Software Version 6. Cells were illuminated with a 488-nm laser line, and the fluorescence was determined using a 530 ± 30 emission filter. The viable population was gated by side scatter/forward scatter evaluation, and the mean fluorescence intensity of duplicate samples was calculated. Mean values of chol-siGFP-treated cells were corrected for background fluorescence by subtracting wild-type cell measurements, and for non-specific treatment effects by normalizing to samples treated with control chol-siRNA but otherwise identical conditions. The gating strategies used are shown in Supplementary Fig. 8.

### Real-time qPCR

HeLa-d1-eGFP or HeLa wild-type cells were incubated with 40 nM chol-siGFP (d1-eGFP), 200 nM chol-siPPIB, or chol-siGAPD (wild type), or control chol-siRNA at the same concentrations, prepared OptiMEM. After 6 h, the medium was removed and cells were washed once with PBS and incubated with complete DMEM supplemented with the indicated drugs for 18 h. At the end of the experiment, samples were washed once with PBS, and RNA extraction was performed using GenElute Mammalian Total RNA Miniprep Kit (Sigma) according to the manufacturer’s protocol. SuperScript III First-Strand Synthesis System (Sigma) was used for complementary DNA synthesis with random hexamer primers, and performed on an Mastercycler EpGradient 5341 thermal cycler (Eppendorf AG, Hamburg, Germany). SYBR Green Jumpstart Taq Readymix (Sigma) was used for qPCR reactions, and performed on a StepOnePlus Real-Time PCR System with MicroAmp Fast 0.1 mL 96-well Reaction Plates (Applied Biosystems, Foster City, CA, USA). *GAPD* or *ACTB* was used as house-keeping genes for normalization. Samples treated with control chol-siRNA with or without any additional drug treatment was used as calibrator samples. Fold differences compared to controls was calculated using the ΔΔC_t_ method. Data were analyzed with StepOne Software v.2.3.

### Microscopy

Two imaging platforms were used, as designated in the text. Relevant acquisition parameters of experiments are given in the corresponding Methods section. Both platforms have heated stage-top incubators and heating inserts (Pecon), Definite focus, CO_2_ control systems, and are operating at 37 °C and 5% CO_2_ during live-cell experiments.

Confocal platform: Inverted AxioOberver Z.1 LSM 710 confocal laser scanning microscope with Airyscan detector (Carl Zeiss AG, Oberkochen, Germany), equipped with a ×63/1.4 Plan-Apochromat oil-immersion, ×40/1.3 EC Plan-Neofluar oil-immersion, ×63/1.2 C-Apochromat Corr water-immersion objective lens (all Zeiss), diode laser 405 nm, Lasos Argon laser 458/488/514 nm, DPSS 561 nm, and HeNe laser 633 nm. The Airyscan detector was used in super-resolution or confocal mode as stated above. The system operates under ZEN 2.1 (black).

Widefield platform: Inverted AxioOberver Z.1 microscope equipped with ×63/1.40 Plan-Apochromat oil-immersion objective lens and Colibri 7 solid state LED light source (Zeiss), MS2000 XY Piezo *Z* stage (Applied Scientific Instrumentation, Euguene, OR, USA), and an ORCA-Flash4.0 V3 Digital CMOS camera (Hamamatsu Photonics, Hamamatsu City, Japan). The system was used in fast acquisition mode (camera, illumination, and stage) using a SVB 1 signal distribution box (Zeiss) and μCon HS Trigger board (PCIxPress). The system operates under ZEN 2.3 (blue). The following multi-bandpass filters were used (Zeiss): Multiband filter set 92 HE LED (E) with triple-band exciter, emitter, and beamsplitter filters: excitation wavelengths 385, 475, and 590 nm, TBS 405, 493, and 610 nm, TBP 425 ± 30, 524 ± 50, and 688 ± 145 nm. Multiband filter set 90 HE LED (E) with quad-band exciter, emitter and beamsplitter filters: excitation wavelengths 385, 475, 555, and 630 nm, QBS 405, 493, 575, and 653 nm, QBP 425 ± 30, 514 ± 30, 592 ± 30, and 709 ± 100 nm. For live-cell experiments, typically 1.1% or 1.2% LED illumination was used with 20-ms camera exposure in two-channel acquisitions, and 5% with 10- or 20-ms camera exposure for three-channel acquisitions. Channels were acquired sequentially for each *z*-plane, to reduce latency between channels in the same *z*-plane. Thirty planes were acquired per *z*-stack with 300-nm interval. Images were acquired for ∼20 min per position, and approximately four to eight time-lapse acquisitions were collected per experiment.

### Widefield microscopy image processing and analysis

An image processing and analysis program with a graphical user interface was developed in MATLAB, and is available at https://github.com/hdurietz/QuantEscape together with documentation and links to example datasets. The program streamlines work with large microscopy datasets, using batch processing and subsets of the total data, to enable efficient object tracking and quantitative analysis of multi-channel live-cell imaging experiments.

In brief, images were exported as raw data 16-bit tiff-files from the image acquisition software, and deconvolved using Huygens Professional Batch processing interface and empirical point spread functions. Maximum intensity projections of deconvolved *z*-stacks were created for inspection and event calling. Approximate coordinates (*x*, *y*, and *t*) of de novo appearing galectin-9 foci were identified in Fiji, and exported together with a cell outline and background ROI. The MATLAB program uses the provided coordinates to create 5D ROIs of single events from deconvolved images, together with image background and cell measurements. The coordinates are also used as a seeding point for automated detection of the time (*t*_0_) and *x*, *y*, and *z* position of the appearing galectin foci. The algorithm will search the complete *z* volume within a user-specified distance from the *xy* seeding point (typically ±6 pixels square offset in *x-* and *y*-direction). The initial search is started 25 frames before the frame of the seeding point. Any object measurements made during the initial search that is greater than the object detection threshold will be confirmed or rejected as the true *t*_0_ in a second step. The object detection threshold is calculated as the mean + 5 × s.d. of all measurements before *t*_0_, and can be modified to achieve the appropriate sensitivity. In the second step, the *x*, *y*, and *z* coordinates at the putative *t*_0_ is used as a new seeding point in the subsequent frame. An object mask is fitted to the maximum intensity within a volume that is now confined in *x*, *y*, and *z* dimension by an object tracking distance (square offset) in *x* and *y* dimension as well as a *z*-offset maximum (typically 12 pixels and 5 *z*-planes, respectively). If this process can be repeated in five consecutive frames after *t*_0_, always yielding intensity measurements above the object detection threshold, the detected object is assumed to be the true galectin recruitment event.

For tracking of detected galectin foci in cells with labeled endosomal compartments, the initial *x*, *y,* and *z* coordinates of the galectin object at *t*_0_ was used for static measurements at typically 20 frames before *t*_0_. From *t*_0_ and on, the object mask was fitted to yield the maximum intensity measurements within the defined *x*, *y*, and *z* object tracking distances as described above. Object measurements were performed within a single plane for all channels, using a 6-pixel (∼600 nm) diameter object mask.

### Software

MATLAB 2018a was used for data analysis as described above. CellProfiler 2.1.1 with customized pipelines was used for galectin puncta quantifications and segmentation of cells and intracellular objects. Microsoft Excel for Mac Version 15.17 was used for calculations and data processing. GraphPad Prism 8 for Mac Version 8.0.1 was used to create figures and perform statistical testing. The Manual Tracking plugin in Icy version 1.6.1.1 was used for tracking vesicles to compare galectin recruitment. Fiji 1.52i was used for analysis and visualization of microscopy images. Brightness and contrast settings were linearly adjusted and kept identical for images intended for comparison. Adobe Photoshop 2020 version 21.0.2 was used for composing and annotating microscopy image figures. Final figures were assembled in Adobe Illustrator 2020 version 24.0.1.

### Statistics

Statistical tests were performed with RStudio Version 1.1.442 using t.test, wilcox.test, and fisher.test, as described in figure legends. Linear regression models and standard curves were fitted with GraphPad Prism version 8.0.1, using least-squares regression and “Sigmoidal, 4PL, X is log(concentration)” interpolation, respectively. Estimated 95% confidence intervals of means were calculated as 1.96 × s.e.m.

### Reporting summary

Further information on research design is available in the Nature Research Reporting Summary linked to this article.

## Supplementary information

Supplementary Information

Peer Review File

Description of Additional Supplementary Files

Supplementary Movie 1

Supplementary Movie 2

Supplementary Movie 3

Supplementary Movie 4

Supplementary Movie 5

Reporting Summary

## Data Availability

The source data underlying all quantitative figures are provided as a Source Data file. All data supporting the findings of this study is available from the corresponding author upon reasonable request.

## Source Data

Source Data

## Code availability

The MATLAB program for vesicle and siRNA tracking is available at https://github.com/hdurietz/QuantEscape with documentation and links to example datasets. Additional MATLAB code used for image analysis is available upon request.
