# Supplementary material for: Imaging small molecule-induced endosomal escape of siRNA
Source: Nat Commun. 2020 Apr 14;11:1809. doi: 10.1038/s41467-020-15300-1 (PMC7156650; doi:10.1038/s41467-020-15300-1)
Supplement: Supplementary file 1 — Supplementary Information [file 41467_2020_15300_MOESM1_ESM.pdf]

Supplementary Information

Imaging small molecule-induced endosomal escape of siRNA

Du Rietz *et al.*

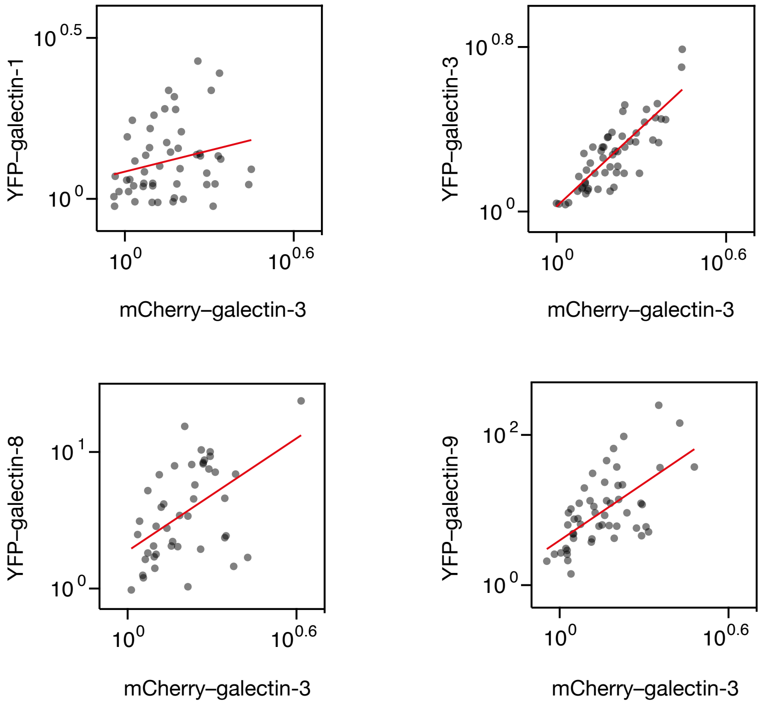

Supplementary Figure 1 | Galectin-3 and -9 response to induced endosomal damage is correlated. Quantification of galectin recruitment from confocal microscopy images of HeLa cells expressing the indicated galectin pairs treated with 10 μM siramesine. Fold recruitment was estimated ~90 s after formation of detectable galectin foci. Red line is linear regression. *N* = 51, 54, 44, 52 events from at least two independent experiments.

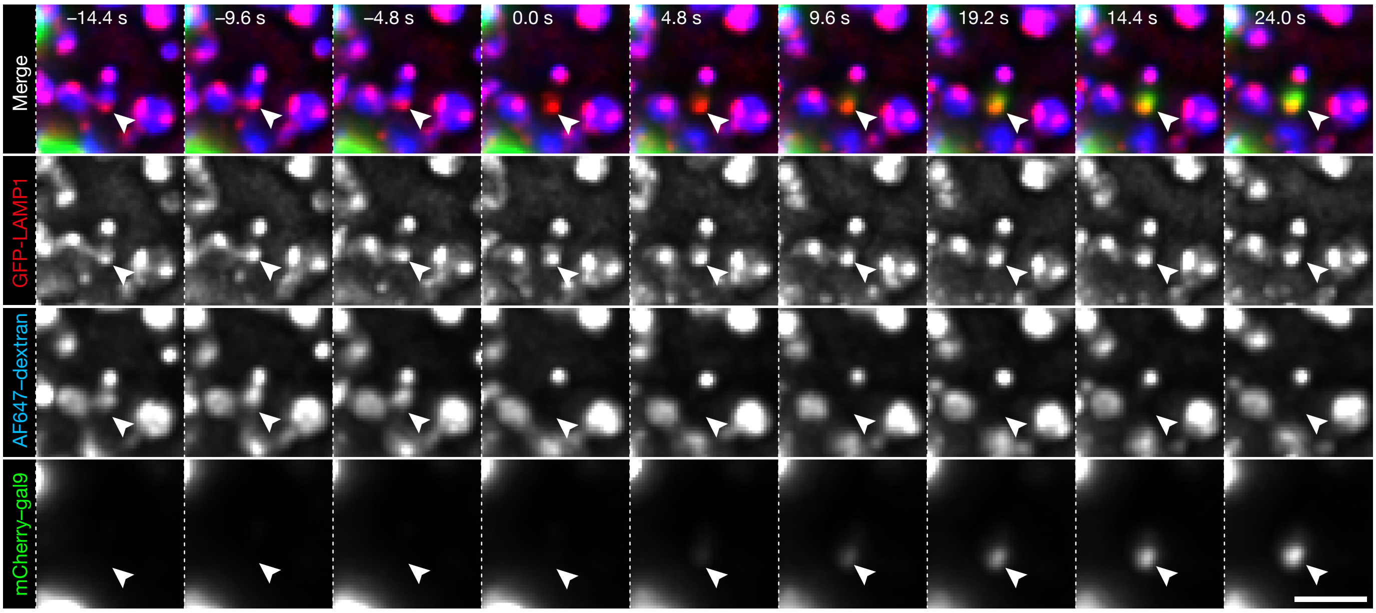

Supplementary Figure 2 | Dextran is released from LAMP1⁺ endosome, followed by galectin-9 recruitment. HeLa cells expressing mCherry–galectin-9 and GFP-LAMP1 were incubated with 200 mg mL^{-1} AF647-labeled dextran, followed by treatment with 60 μM chloroquine. Images were acquired with a widefield microscope. Arrows indicate the releasing endosome. *t* = 0 is the first frame with detectable galectin-9 recruitment. Images are representative of four independent experiments. Maximum intensity projections of *z*-stacks are shown. Scale bar, 2 μm.

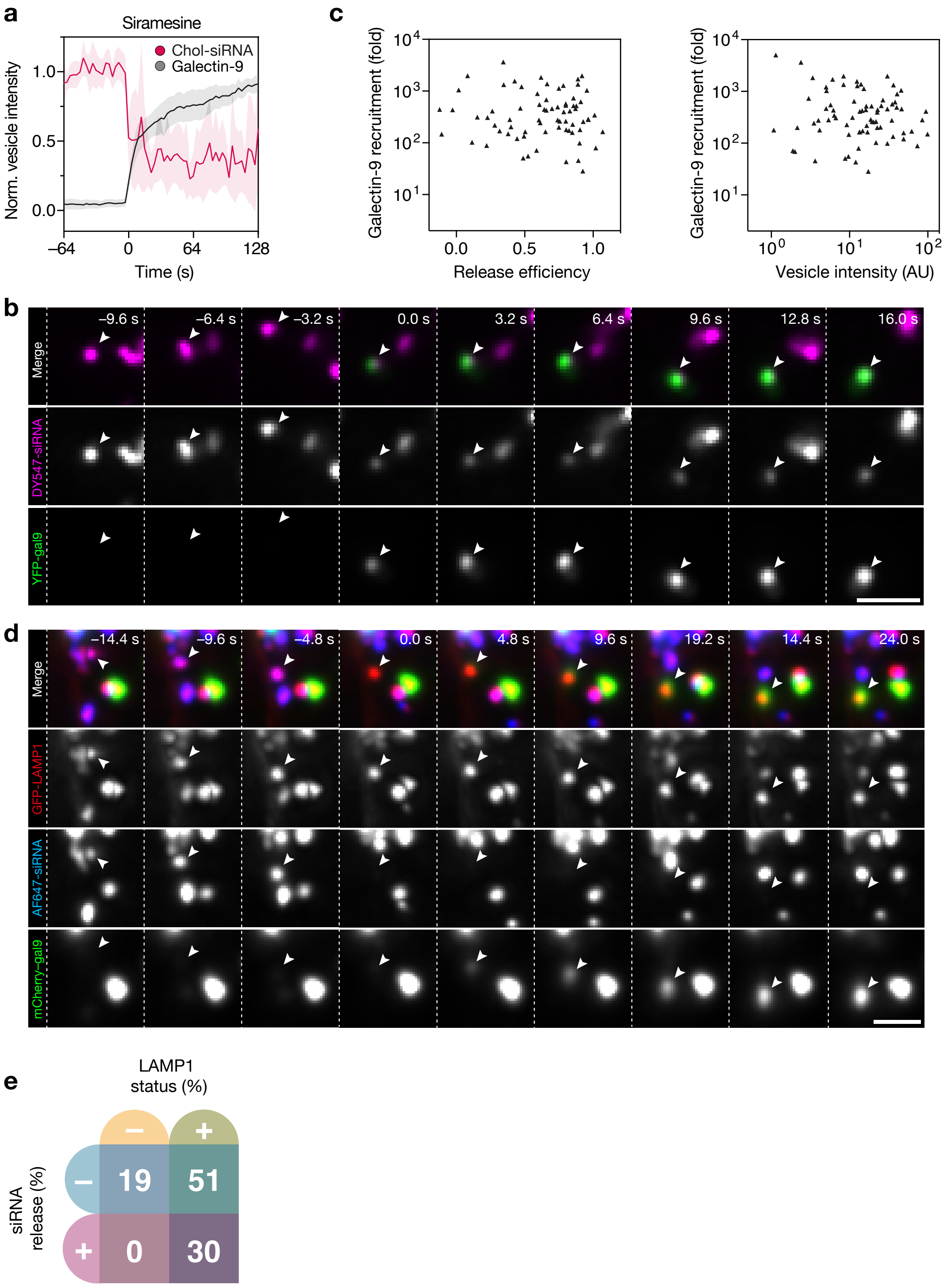

Supplementary Figure 3 | Cholesterol-siRNA is released from LAMP1$^+$ compartments. HeLa cells expressing YFP–galectin-9 were incubated with 200 nM cholesterol-siRNA for 6 h followed by treatment with 60 μM chloroquine or 10 μM siramesine during widefield microscopy. (**a**) Quantification of chol-siRNA release during siramesine treatment, by single-vesicle tracking. Line is mean, shade is 95% CI. Traces are aligned so that galectin recruitment is first detected at t = 0. N = 10 vesicles from two independent experiments. (**b**) Galectin-9 fold recruitment and cholesterol-siRNA release efficiency or vesicle intensity. N = 72, 76 vesicles from two independent experiments. (**c**) Siramesine treatment. Arrows indicate the releasing endosome. Images are representative of three independent experiments. Scale bar, 2 μm. (**d**) HeLa cells expressing mCherry–galectin-9 and GFP–LAMP1 were incubated with 200 nM AF647-labeled chol-siRNA for 6 h followed by chloroquine treatment during widefield microscopy. Arrows indicated the releasing endosome. Images are representative of 16 events from two independent experiments. t = 0 is the first frame with detectable galectin-9 recruitment. Scale bar, 2 μm. (**e**) The frequency of cholesterol-siRNA release from LAMP1$^+$ or LAMP1$^-$ structures was determined by manual classification. N = 53 events from two independent experiments.

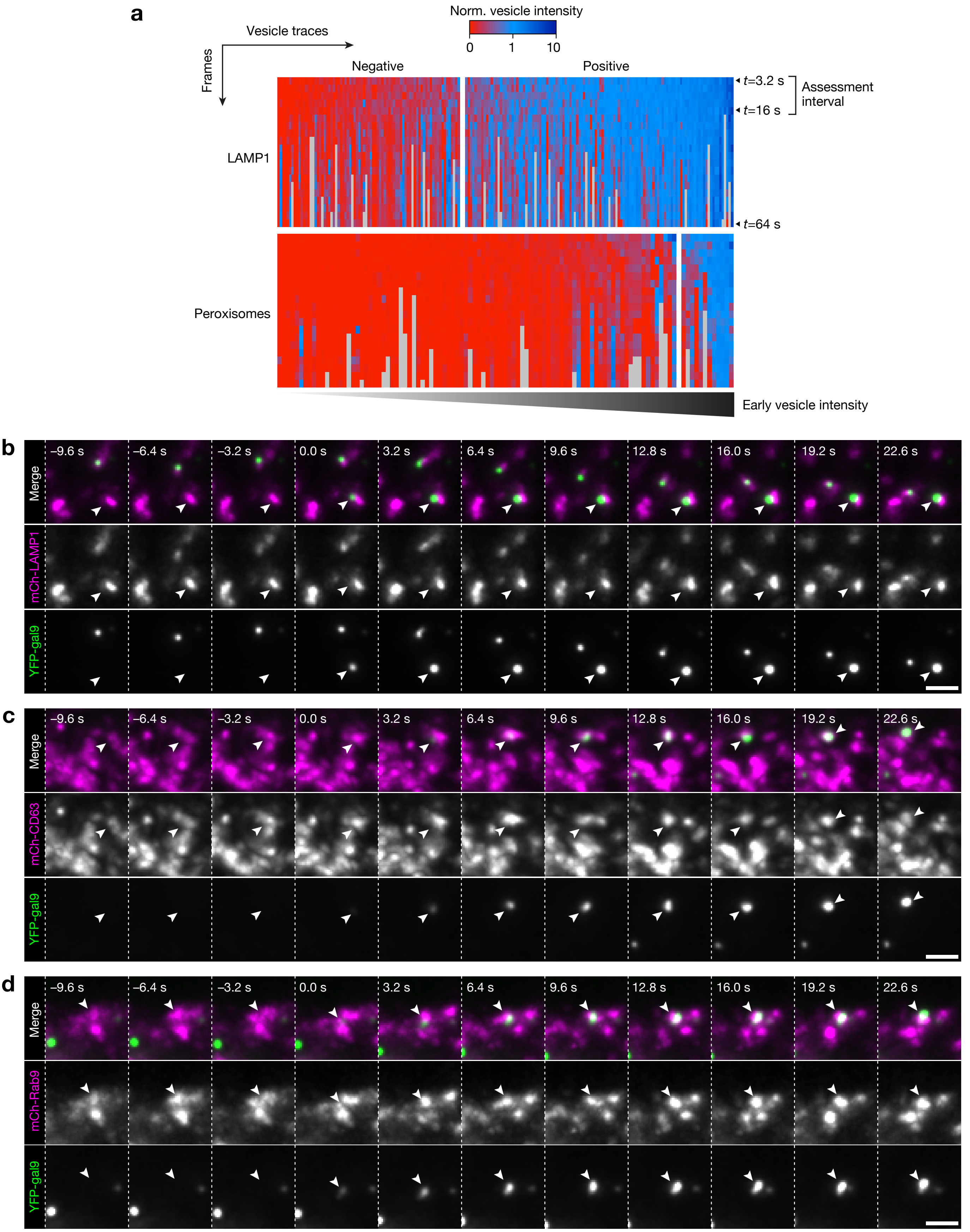

Supplementary Figure 4 | Damaged vesicles display several late endosomal markers. (**a**) HeLa cells expressing YFP-galectin-9 and mCherry-tagged LAMP1 or peroxisome marker (PTS1) were treated with 1 mM LLOMe during widefield live-cell microscopy. *De novo* induced galectin foci were tracked in 4D, and the marker fluorescence intensity of damaged objects were assessed between ~3 and 16 s after detectable galectin recruitment. Heatmaps show the normalized fluorescence intensity of indicated endosomal and organelle markers on disrupted vesicles. Single-vesicle traces are organized in columns. Rows represents measurements between ~3 and 64 s after detectable galectin-9 recruitment, from top to bottom. t = 0 is the first frame with detectable galectin-9 recruitment. Traces were classified as negative (left subdivisions) or positive (right subdivisions) for the respective endosomal marker, and sorted by the mean initial trace intensity for visualization. N = 195 and 105 events from two independent experiments. (**b**-**d**) HeLa cells expressing YFP-galectin-9 and the indicated mCherry-tagged marker were treated with 10 μM siramesine during widefield live-cell microscopy, and galectin-9 recruitment to marker-labeled structures was evaluated. Arrows indicate galectin-9$^+$ vesicles. t = 0 is the first frame with detectable galectin-9 recruitment. Images are respresentative of at least two independent experiments. Scale bars, 2 μm. (**b**) mCherry-LAMP1; (**c**) mCherry-CD63; (**d**) mCherry-Rab9.

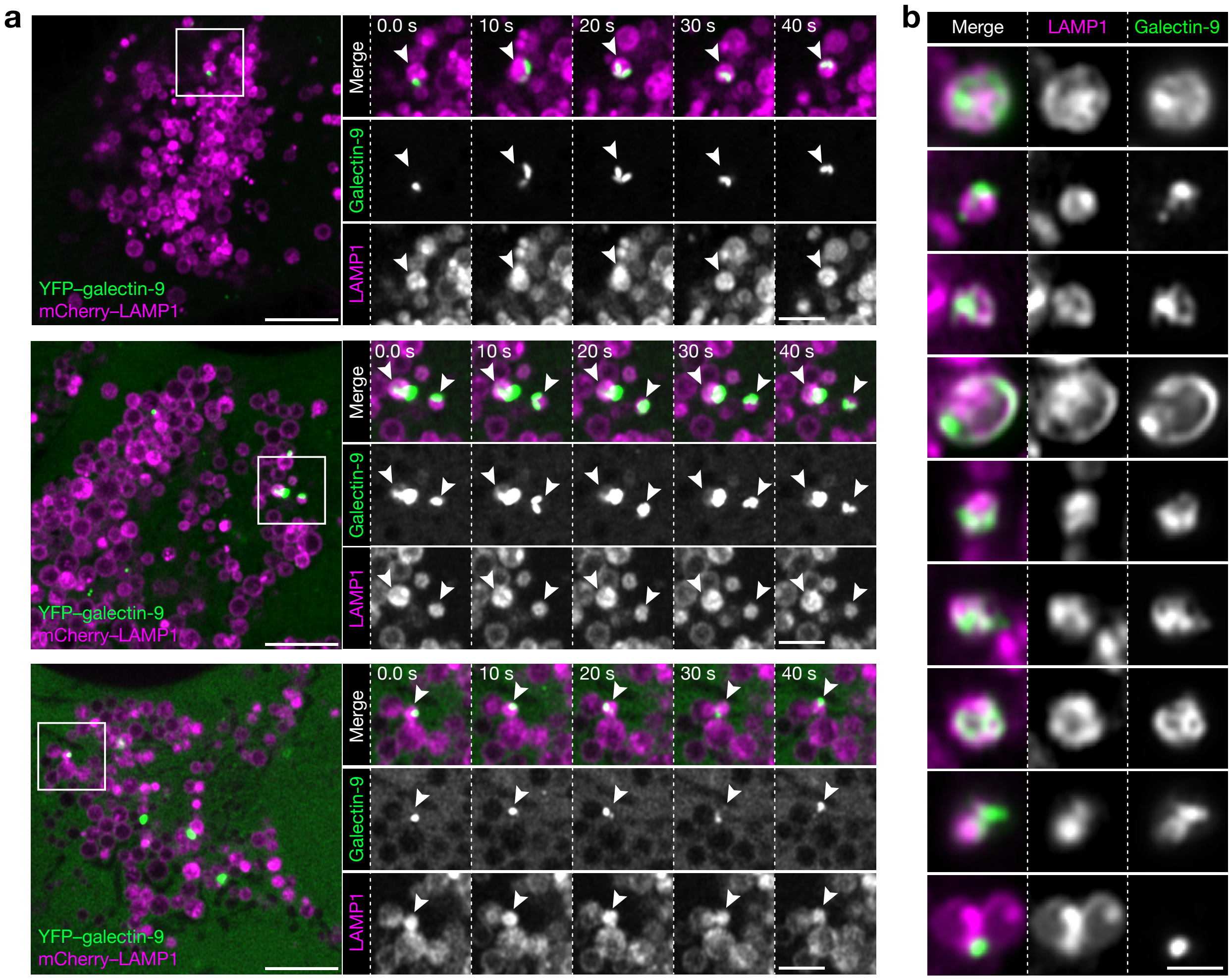

Supplementary Figure 5 | Galectin-9 is recruited to LAMP1⁺ vesicles during small molecule treatment. Airyscan confocal microscopy images of cells expressing mCherry–LAMP1 (**a**) during treatment with 100 μM chloroquine (live-cell), and (**b**) after 12 h treatment with 60 μM chloroquine followed by fixation. Arrows indicate galectin-9 foci on LAMP1⁺ vesicles. Images are representative of two independent experiments. *t* = 0 is the start of image acquisition. Scale bars, (a) 5 μm; details, 2 μm; (b) 1 μm.

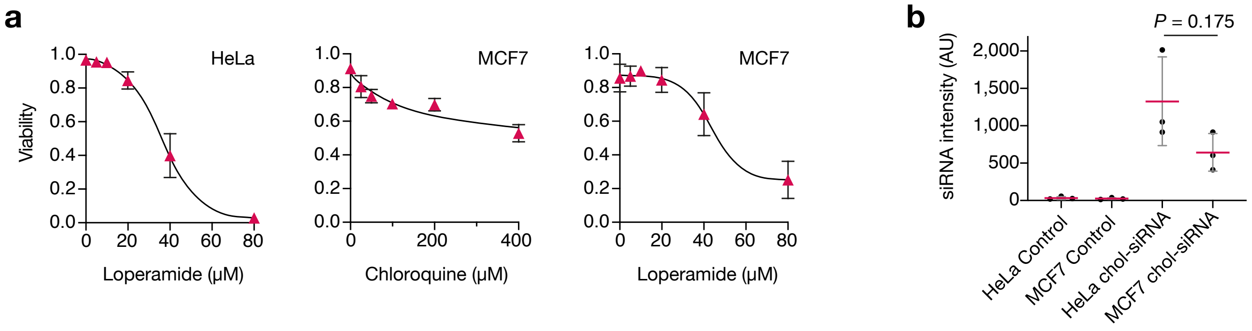

Supplementary Figure 6 | Loperamide treatment is well tolerated at concentrations inducing significant membrane damage. (**a**) Cell viability was evaluated after 24 h drug treatment, by propidium iodide staining and flow cytometry. Mean ± s.d. $N = 3$ independent experiments. (**b**) HeLa and MCF7 cells were incubated with 200 nM chol-siRNA for 6 h, and quantitative evaluation of the mean siRNA content per cell was performed using confocal microscopy. Mean ± s.d. $N = 3$ independent experiments. Two-tailed Student's *t*-test.

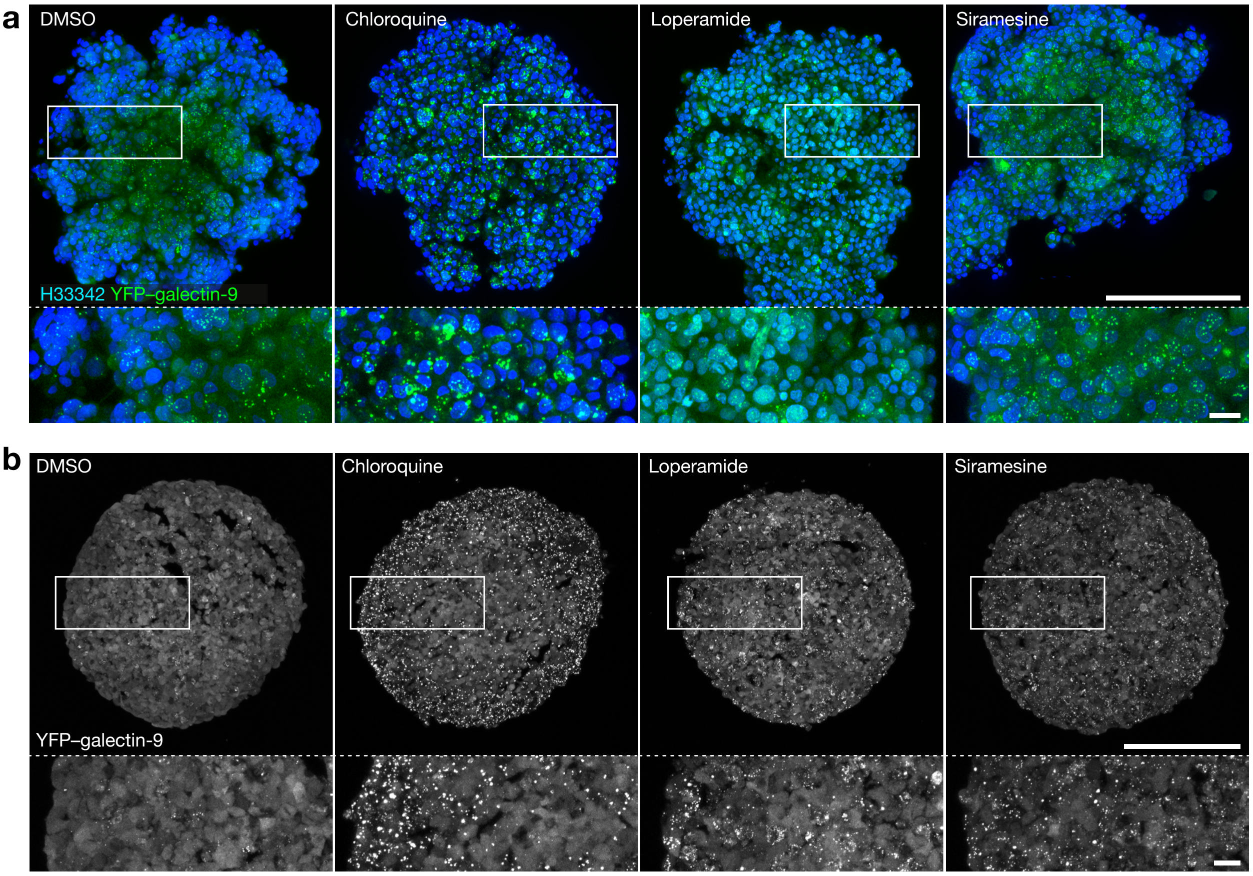

Supplementary Figure 7 | Small molecule drugs trigger endosomal disruption throughout tumor cell spheroids. (**a**) MCF7-galectin-9–YFP cell spheroids incubated with 100 μM chloroquine, 20 μM loperamide or 10 μM siramesine for 20 h imaged with confocal microscopy after optical clearing. Maximum intensity projections of 90-μm *z*-stacks are shown. Images are representative of two independent experiments with at least three spheroids per condition. Scale bars are 200 μm; details 20 μm.(**b**) HeLa-galectin-9–YFP cell spheroids incubated with 100 μM chloroquine, 20 μM loperamide or 10 μM siramesine for 20 h imaged with confocal microscopy after cryosectioning of the central region. Maximum intensity projections of 15-μm *z*-stacks are shown. Images are representative of three independent experiments with at least three spheroids per condition. Scale bars are 200 μm; details 20 μm.

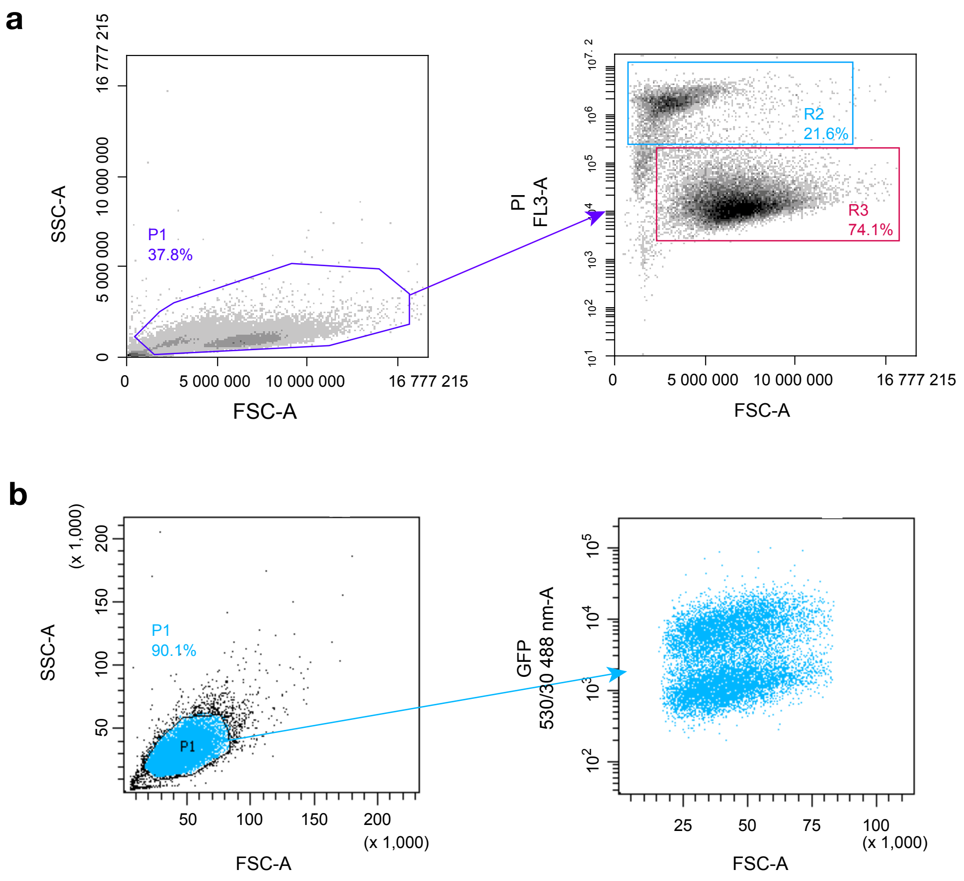

Supplementary Figure 8 | Flow cytometry gating strategies for viability and eGFP knockdown experiments. (**a**) Gating strategy used to estimate cell viability in Fig. 1d,e and Supplementary Fig. 6a. The fraction of cells in R3 compared to all gated cells was regarded as the viable cell fraction. (**b**) Gating strategy used to estimate eGFP knockdown, as presented in Fig. 2c-e; Fig. 3a,c,d; Fig. 8e and Fig. 9c. The mean eGFP fluorescence intensity of the gated cells (P1) was used to estimate target knockdown, as described in the Methods section.
