# Supplementary material for: Imaging small molecule-induced endosomal escape of siRNA
Source: Nat Commun. 2020 Apr 14;11:1809. doi: 10.1038/s41467-020-15300-1 (PMC7156650; doi:10.1038/s41467-020-15300-1)
Supplement: Supplementary file 3 — Description of Additional Supplementary Files [file 41467_2020_15300_MOESM3_ESM.pdf]

Description of Additional Supplementary Files

Supplementary Movie 1 | Formation of galectin-9 foci during chloroquine treatment. HeLa cells expressing YFP-galectin-9 was monitored with confocal microscopy during 24 h, while treated with 60 µM chloroquine. Time-lapse recording is representative of three independent experiments. Maximum intensity projections of *z*-stacks are shown.

Supplementary Movie 2 | Endosomal release of chol-siRNA. HeLa cells expressing YFP-galectin-9 were incubated with 200 nM DY547-labeled chol-siRNA and treated with 60 µM chloroquine while acquiring images with a widefield microscope. White circles indicate the releasing endosome. Images are maximum intensity projections of *z*-stacks. The sequence is representative of 78 release events from two independent experiments.

Supplementary Movie 3 | Chol-siRNA is released from LAMP1$^+$ endosome. HeLa cells expressing mCherry-galectin-9 and GFP-LAMP were incubated with 200 nM AF647-labeled chol-siRNA and treated with 60 µM chloroquine while acquiring images with a widefield microscope. White circles indicate the releasing endosome. Images are maximum intensity projections of *z*-stacks. The sequence is representative of 16 release events from two independent experiments.

Supplementary Movie 4 | Rab5 is recruited to vesicles disrupted by chloroquine. HeLa cells expressing YFP-galectin-9 and mCherry-Rab5 were treated with 60 µM chloroquine while acquiring images with a widefield microscope. White circles indicate *de novo* formation of galectin-9 foci. Images are maximum intensity projections of *z*-stacks. Three events are shown, representative of 44 events from two independent experiments.

Supplementary Movie 5 | Small molecules induce widespread vesicle damage in tumor spheroids. Volumetric rendering of HeLa-galectin-9–YFP spheroids, also shown in Fig. 7a. Spheroids were incubated with 100 µM chloroquine, 20 µM loperamide or 10 µM siramesine for 20 h, and imaged with a confocal microscope after optical clearing. *Z*-stacks are 45 µm. Images are representative of three independent experiments with at least three spheroids per condition.
