# Supplementary material for: Imaging small molecule-induced endosomal escape of siRNA
Source: Nat Commun. 2020 Apr 14;11:1809. doi: 10.1038/s41467-020-15300-1 (PMC7156650; doi:10.1038/s41467-020-15300-1)
Supplement: Supplementary file 9 — Reporting Summary [file 41467_2020_15300_MOESM9_ESM.pdf]

nature research

Corresponding author(s): Anders Wittrup

Last updated by author(s): Feb 25, 2020

Reporting Summary

Nature Research wishes to improve the reproducibility of the work that we publish. This form provides structure for consistency and transparency in reporting. For further information on Nature Research policies, see Authors & Referees and the Editorial Policy Checklist.

Statistics

For all statistical analyses, confirm that the following items are present in the figure legend, table legend, main text, or Methods section.

n/a	Confirmed	
☐	☒	The exact sample size (*n*) for each experimental group/condition, given as a discrete number and unit of measurement
☐	☒	A statement on whether measurements were taken from distinct samples or whether the same sample was measured repeatedly
☐	☒	The statistical test(s) used AND whether they are one- or two-sided<br>*Only common tests should be described solely by name; describe more complex techniques in the Methods section.*
☒	☐	A description of all covariates tested
☐	☒	A description of any assumptions or corrections, such as tests of normality and adjustment for multiple comparisons
☐	☒	A full description of the statistical parameters including central tendency (e.g. means) or other basic estimates (e.g. regression coefficient) AND variation (e.g. standard deviation) or associated estimates of uncertainty (e.g. confidence intervals)
☐	☒	For null hypothesis testing, the test statistic (e.g. *F*, *t*, *r*) with confidence intervals, effect sizes, degrees of freedom and *P* value noted<br>*Give P values as exact values whenever suitable.*
☒	☐	For Bayesian analysis, information on the choice of priors and Markov chain Monte Carlo settings
☒	☐	For hierarchical and complex designs, identification of the appropriate level for tests and full reporting of outcomes
☒	☐	Estimates of effect sizes (e.g. Cohen's *d*, Pearson's *r*), indicating how they were calculated

Our web collection on statistics for biologists contains articles on many of the points above.

Software and code

Policy information about availability of computer code

Data collection	ZEN 2.3 (blue), ZEN 2.1 (black), BD Accuri C6 Software Version 1.0.264.21, BD FACSDiva Software Version 6. StepOne Software v.2.3.
Data analysis	Huygens Professional for Win64 Compute engine 17.04.0p6 64b, MATLAB 2018a, CellProfiler 2.1.1, Microsoft Excel for Mac Version 15.17, Graphpad Prism 8 for Mac Version 8.0.1, Fiji 1.52i, RStudio Version 1.1.442 with stat Package (v3.4.4) with t.test, wilcox.test and fisher.test. Adobe Photoshop 2020 version 21.0.2 and Adobe Illustrator version 24.0.1. Custom MATLAB code was used for endosomal escape and vesicle analysis, available at https://github.com/hdurietz/QuantEscape.

For manuscripts utilizing custom algorithms or software that are central to the research but not yet described in published literature, software must be made available to editors/reviewers. We strongly encourage code deposition in a community repository (e.g. GitHub). See the Nature Research guidelines for submitting code & software for further information.

Data

Policy information about availability of data

All manuscripts must include a data availability statement. This statement should provide the following information, where applicable:

- Accession codes, unique identifiers, or web links for publicly available datasets
- A list of figures that have associated raw data
- A description of any restrictions on data availability

All data is available upon request. Source Data for all quantitative Figures and Supplementary figures is provided as Supplementary Information. Custom MATLAB code for endosomal escape and vesicle analysis is publicly available as stated above. Additional MATLAB code is available upon request.

Field-specific reporting

Please select the one below that is the best fit for your research. If you are not sure, read the appropriate sections before making your selection.

☒ Life sciences ☐ Behavioural & social sciences ☐ Ecological, evolutionary & environmental sciences

For a reference copy of the document with all sections, see nature.com/documents/nr-reporting-summary-flat.pdf

Life sciences study design

All studies must disclose on these points even when the disclosure is negative.

Sample size	No formal sample-size calculations were performed. For statics on single vesicle and single cells, data was collected for as many data points (vesicles or cells) as technically possible. The experimental setup for single vesicle characterization was validated initially as described in the manuscript with a positive and negative control dataset (LLOMe vs LAMP1 and peroxisomes). Using this dataset, parameter estimates appeared stable upon addition of new data after 20 events. For dynamics characterization and most key experiments we aimed for at least twice this number of events.
Data exclusions	For vesicle analysis in microscopy experiments, non-trackable or quantifiable events were excluded from further analysis. For compartment identity mapping, signal-to-noise filtering with respect to marker expression was equally performed for all groups during data analysis. Given that these experiments were performed on cells with transient (and thus variable) marker expression, some cells invariably had no or very low marker expression. To avoid manual and potentially subjective exclusion of these cells, all cells and detected events were registered and subsequently those cells with a non-usuable expression level (average cell object signal-to-noise ratio compared to the image background below 50, calculated as described in the Methods section) were excluded. This strategy was first validated using the positive and negative control data set (LLOMe vs LAMP1 and peroxisomes) and applied consistently for all compartment analysis experiments. Apart from the analysis workflow described above, no data was excluded from analysis.
Replication	All results were replicated in at least two independent experiments, as described in the text. All reported results were replicable.
Randomization	No sample randomization or allocation strategy was used. Only in vitro experiments were performed, where randomization of cell samples is typically not practical.
Blinding	No blinding was applied during experiments or data analysis for practical feasibility given the nature of the project. However, all quantitative cell and vesicle analysis was performed using observer independent algorithms. Further, for all other experiments, all data was included in analysis and handled without subjective analysis criteria, limiting the need for formal blinding.

Reporting for specific materials, systems and methods

We require information from authors about some types of materials, experimental systems and methods used in many studies. Here, indicate whether each material, system or method listed is relevant to your study. If you are not sure if a list item applies to your research, read the appropriate section before selecting a response.

Materials & experimental systems

n/a	Involved in the study
☐	☒ Antibodies
☐	☒ Eukaryotic cell lines
☒	☐ Palaeontology
☒	☐ Animals and other organisms
☒	☐ Human research participants
☒	☐ Clinical data

Methods

n/a	Involved in the study
☒	☐ ChIP-seq
☐	☒ Flow cytometry
☒	☐ MRI-based neuroimaging

Antibodies

Antibodies used	Rat anti-galectin-3 antibody (M3/38, PMID: 6173426), produced in-house in the lab of H. Leffler.<br>Cy3-conjugated AffiniPure Donkey anti-Rat IgG secondary antibodiy (Code 712-165-150, Lot 68682) were from Jackson ImmunoResearch Laboratories Inc., West Grove, PA, USA.
Validation	The M3/38 antibody has been extensively used over decades and is highly validated. The in-house produced batch was validated using western blot against a dilution series of purified galectin-3.

Eukaryotic cell lines

Policy information about cell lines

Cell line source(s)	HeLa and MCF7 cell lines were purchased from the American Type Culture Collection (ATCC).
Authentication	According to ATCC standards, originally including STR profiling and isoenzyme verification, and by continuous cell morphology and growth monitoring.
Mycoplasma contamination	All cell lines were repeatedly tested and confirmed to be free from Mycoplasma infection.
Commonly misidentified lines (See ICLAC register)	Commonly misidentified cell lines were not used.

Flow Cytometry

Plots

Confirm that:

☒ The axis labels state the marker and fluorochrome used (e.g. CD4-FITC).

☒ The axis scales are clearly visible. Include numbers along axes only for bottom left plot of group (a 'group' is an analysis of identical markers).

☒ All plots are contour plots with outliers or pseudocolor plots.

☒ A numerical value for number of cells or percentage (with statistics) is provided.

Methodology

Sample preparation	Samples were prepared from in vitro cultures by trypsin treatment. For experiments with multiple read-out timepoints, samples were fixed with 4% PFA for 20 min on ice, keept at 4°C, and analysed at the end of experiments. Details are provided in the methods section.
Instrument	BD Accuri C6 Cytometer. Becton Dickinson, Franklin Lakes, NJ, USA.<br>BD FACSAria IIu Cell Sorter, Becton Dickinson.
Software	BD Accuri C6 Software Version 1.0.264.21.<br>BD FACSDiva Software Version 6.
Cell population abundance	No cell sorting experiments were performed. The number of cells analyzed was dependent on the experiment, and were typically at least 5,000 cells. No purity assessment was performed.
Gating strategy	For viability experiments, the viable and apoptotic popluations were gated separately in FL3/FSC scatter plots. For EGFP knockdown, viable cells were gated in FSC/SSC scatter plots ,and all gated cells were used in analysis. Examples of the gating strategies used are provided in Supplementary Fig. 8.

☒ Tick this box to confirm that a figure exemplifying the gating strategy is provided in the Supplementary Information.
