# Supplementary material for: Imaging small molecule-induced endosomal escape of siRNA
Source: Nat Commun. 2020 Apr 14;11:1809. doi: 10.1038/s41467-020-15300-1 (PMC7156650; doi:10.1038/s41467-020-15300-1)
Supplement: Supplementary file 2 — Peer Review File [file 41467_2020_15300_MOESM2_ESM.pdf]

Reviewers' comments:

Reviewer #1 (Remarks to the Author):

Rietz used live-cell microscopy and cytosolic galectin-9 as a sensor of membrane damage, to probe fundamental properties of endosomal escape of cholesterol conjugated siRNA induced by various endosome-disrupting small molecules. Authors demonstrate efficient release of ligand-conjugated siRNA from vesicles damaged by small molecules, enhancing target knockdown up to ~47-fold in tumor cells. Still, mismatch between siRNA containing and drug-targeted endo/lysosomal compartments limits siRNA activity improvement. This strategy to characterize endosomal escape presented here will be widely applicable, facilitating future efforts to improve delivery of siRNA and other nucleic acid-based therapeutics. This is an elegant study with a very well written paper and I think one of the best papers this year I have reviewed with regards to endosomal escape. I have some minor critiques, but I strongly support publication of this work.

1. In Fig. 3 it is shown that even though chol-siRNA are present in LAMP1 positive vesicles, there is a window of opportunity for escape prior to reaching to the lysosome. But supplementary fig. 3d, e suggest that the siRNA release is from LAMP positive vesicles, and therefore post treatment of chloroquine after siRNA was delivered should have worked. Please explain this discrepancy.

2. The other question is regarding the cell types differences that are observed in these studies. Fig 8. shows that there is comparatively large amount of galectin-9 positive foci in MCF-7 cells as compared to HeLa cells, yet the knock down remains limited. Is the uptake in MCF-7 and Hela equivalent or low amount of internalization corresponds to this decrease in knock down. If uptake is equal, perhaps these are downstream events but it is important to show that the overall uptake in HeLa and MCF-7 are equivalent to make comparisons on the silencing activity. The escape kinetics and the foci number are similar, but the lack of silencing is a little confusing. Please explain.

3. Fig. 6 is just excellent, and I think very elegantly shows spatiotemporal dynamics of various endosomal markers. The appearance of Rab5 on galectin positive endosomes is intriguing. Since the escape by Wittrup et al., in earlier papers have shown to be Rab5-dependent. Is it possible these are some remnants of Rab5 positive endosomes that have undergone escape and have not shed Rab5 rather than it being recruited again after the endosome breaks,. The other issue is in the figures and movies, it seems like lot of conclusions are derived from appearance of a marker and its localization with Galectin, however since in many cases not a complete co-localization is observed, one might surmise that these are multiple vesicles or a fusion event of different hybrids vesicles that are observed and the disappearance of Rab5 might be due to two vesicles in close proximity trying to fuse and perhaps failing to do so. Is the endosomal mapping thus representative of a single endosome or perhaps multiple endosomes in close proximity containing different subset of markers and therefore truly representative of an event at single vesicular level.

4. A minor comment is that in Fig. 1 DMSO seems to do silencing on its own and in next figures that kind of decrease is not seen. This is again a discrepancy that needs to be cleared. Also, using a scrambled control in some of these studies will improve the manuscript, especially that the effects are not due to off targeting.

5. Finally, a recent paper suggested that Gal-8 can act as endosomal sensor for polymers for gene delivery in-vitro and in-vivo (ACS Nano 2019, Duvall et al.). Can the authors explain whether this can be due to difference in delivery system and if so whether different delivery systems might require different sensors or can galectin-9 serve as a universal sensor and whether has relevanve for studying trafficking in animal models.

6. Do the observe change in shape, size of the vesicles that show higher endosomal escape?

6. Finally, I strongly support publication!! Well done!!

Reviewer #2 (Remarks to the Author):

This study supported lots of experiments to elucidate mechanisms of endosomal escape of cholesterol-conjugated siRNA induced by various endosome-disrupting small molecules. A large of data and relevant statistical analysis are provided in this paper. It is an interesting work and valuable for mechanism study on endosomal escape. However, there are still some confusions and list as following:

1. The authors mentioned that galectins could respond to endosomal membrane disruption and relocate to damaged vesicles during lipid-mediated endosomal escape, in that way, can galectins be used as a sensor of membrane damage in other factors mediated endosomal escape as well? Or is there any other substance that can be used to probe endosomal escape?
2. Why choose cholesterol-conjugated siRNA instead of other siRNA nanocarrier delivery systems? How about the effects of small molecules induced endosomal escape on LNP-based or SNALP-based siRNA delivery?
3. Why choose siramesine and amitriptyline as membrane-destabilizing small molecules? Is there any other cationic small molecules also have the properties to facilitate endosomal escape?
4. What factors could possibly affect which kind of siRNA is accumulated in which types of intracellular compartments? For example, is it influenced by the sequence of siRNAs? Maybe by further understanding of this, we can better solve the mismatch between siRNA-containing and drug-targeting compartments which limits the siRNA activity improvement.

Response to referees' comments

We first want to thank both reviewers for their time and effort in providing highly pertinent and constructive review comments. Below is a point-by-point response to the issues raised by the reviewers. Where we have judged it appropriate, we have updated and clarified the manuscript. We have also included additional data and a discussion on the uptake of cholesterol-siRNA in MCF7 cells which we believe has substantially solidified the conclusions regarding point 2 by Reviewer #1.

Reviewer #1

1. *In Fig. 3 it is shown that even though chol-siRNA are present in LAMP1 positive vesicles, there is a window of opportunity for escape prior to reaching to the lysosome. But supplementary fig. 3d, e suggest that the siRNA release is from LAMP positive vesicles, and therefore post treatment of chloroquine after siRNA was delivered should have worked. Please explain this discrepancy.*

This is a very good point, and we did not formulate our line of thought clearly in the manuscript. The release of siRNA is indeed occurring from LAMP1$^+$ vesicles. However, Fig. 3e shows that the precise timing of the drug addition to the cells is not cruicial to the knockdown improvement. Adding siRNA and chloroquine simultaneously seems to result in the same degree of knockdown enhancement as delaying the addition of chloroquine until 6 h after adding the siRNA. This is what we were referring to as the "window of opportunity". However, delaying th addition of chloroquine 12h and thus exposing the cells in this experiment to only 12h of drug treatment abrogated the knockdown improvement. Since it takes several hours for chloroquine to trigger extensive membrane damages, it seems a certain amount of time of substantial membrane damages are required to achieve knockdown improvement. We have clarified our reasoning on the time of drug-induced siRNA release in the text.

2. *The other question is regarding the cell types differences that are observed in these studies. Fig 8. shows that there is comparatively large amount of galectin-9 positive foci in MCF-7 cells as compared to HeLa cells, yet the knock down remains limited. Is the* uptake *in MCF-7 and Hela equivalent or low amount of internalization corresponds to this decrease in knockdown? If uptake is equal, perhaps these are downstream events but it is important to show that the overall uptake in HeLa and MCF-7 are equivalent to make comparisons on the silencing activity. The escape kinetics and the foci number are similar, but the lack of silencing is a little confusing. Please explain.*

Differences between HeLa and MCF7 with regard to the number of galectin-9 foci is due to i) MCF7 cells have more intranuclear galectin-9 foci than HeLa cells, and ii) MCF7 are larger than HeLa cells (~1.5 fold average maximum area per cell, and >2 fold larger volume). The first observation explains the difference at baseline (control), and the second explain why MCF7 have more galectin-9 foci. As the quantifications included the entire cell volume, size-differences play an important role.

The comment regarding a possible difference in siRNA uptake between the cell lines is highly relevant. Although uptake was seemingly efficient in both cell lines, we did not evaluate this experimentally. To rule out that this is an important factor explaining the lower knockdown enhancement in MCF7 cells, we have performed an additional siRNA internalization experiment, quantitatively evaluating the levels of siRNA in HeLa and MCF7 cells. SiRNA uptake in MCF7 cells is approximately half of that in HeLa, likely contributing to the lower knockdown improvement from drug treatments to some extent. However, this cannot in itself explain the vastly lower knockdown improvement seen with in particular loperamide.

As the reviewer states, the release kinetics are similar in MCF7 and HeLa. However, we also observed a lower hit-rate of siRNA-containing vesicles in MCF7. In addition, due to size differences, the amount of siRNA needed to generate equivalent biological effects can be assumed to be higher in MCF7. This is the two main reasons we think underlies the observed difference. The difference in hit-rate could in turn be influenced by multiple factors, including sorting and trafficking of cholesterol-siRNA (*e.g.* lysosomal routing versus recycling), and the possibility of small molecule to target a different spectrum of late endosomal vesicles (LE, endolysosomes or mature lysosomes).

We have updated the manuscript with a clarification of these points.

3. (a) *Fig. 6 is just excellent, and I think very elegantly shows spatiotemporal dynamics of various endosomal markers. The appearance of Rab5 on galectin positive endosomes is intriguing. Since the escape by Wittrup et al., in earlier papers have shown to be Rab5-dependent. Is it possible these are some remnants of Rab5 positive endosomes that have undergone escape and have not shed Rab5 rather than it being recruited again after the endosome breaks?*

In many of the cases with chloroquine, we observe a fast increase of Rab5 to the damaged object. This implies an active Rab5 recruitement after membrane disruption. With siramesine and some cases with chloroquine, the vesicle seems to maintain a constant Rab5 signal after the damage. Since these structures are not marked by galectin before our observation of its recruitment, it is not likely that it was previously damaged. However, there is a possibility that some of these Rab5$^+$ events represent markers located in LE or lysosomes, being degraded. Future more detailed studies with multiple markers in the same cells would be needed to fully elucidate this.

3. (b) *The other issue is in the figures and movies, it seems like lot of conclusions are derived from appearance of a marker and its localization with Galectin, however since in many cases not a complete co-localization is observed, one might surmise that these are multiple vesicles or a fusion event of different hybrids vesicles that are observed and the disappearance of Rab5 might be due to*

two vesicles in close proximity trying to fuse and perhaps failing to do so. Is the endosomal mapping thus representative of a single endosome or perhaps multiple endosomes in close proximity containing different subset of markers and therefore truly representative of an event at single vesicular level.

The point made is highly relevant. The observation of lack of complete colocalization between galectin and the vesicle is intriguing and challenging from a quantification perspective. As the reviewer speculates, we too suspect that the binding of galectin is influenced by some biological processes which can explain the observed pattern. At the current time, the spatial resolution of our live-cell microscopy system while performing these experiments does not allow us to gain more insight into these processes. We did however do some Airyscan Confocal microscopy experiments of both live cells (Fig. 7c and Supplementary Fig. 4b) and fixed, where the higher resolution images confirm this incomplete or off-center localization of galectin-9 with respect to the labeled vesicles. Thus, the incomplete colocalization is a biological phenomenon and not an imaging artefact.

Since we are primarily evaluating the prescence of endosomal markers up until 16 s after the damage event, we try to make the data as representative of the "vesicle state at damage" as possible and spurious vesicles in proximity will not give raise to a false-positive signal. We see that indeed most vesicles seem to maintain their markers throughout the total ~2 min tracks. Some vesicles however, with Rab5 for example, seems to lose the marker relatively shortly after the damage. If this complex pattern represents vesicles trying to fuse, being damaged and then separates, or a single vesicle being damaged and then splits into two separate vesicles, or some other process, is still a completely open question. Again, we hope future studies with even higher spatiotemporal resolution can shed light on this.

4. *A minor comment is that in Fig. 1 DMSO seems to do silencing on its own and in next figures that kind of decrease is not seen. This is again a discrepancy that needs to be cleared. Also, using a scrambled control in some of these studies will improve the manuscript, especially that the effects are not due to off targeting.*

The cells in th figure labeled with DMSO are also incubated with siRNA at the concentrations indicated. Generally, all treatments with active siRNA are compared to negative control siRNA and knockdown expressed relative to the negative control cells.

5. *Finally, a recent paper suggested that Gal-8 can act as endosomal sensor for polymers for gene delivery in-vitro and in-vivo (ACS Nano 2019, Duvall et al.). Can the authors explain whether this can be due to difference in delivery system and if so whether different delivery systems might require different sensors or can galectin-9 serve as a universal sensor and whether has relevanve for studying trafficking in animal models.*

Both galctin-8 and -9 are efficient sensors of membrane damage as shown in multiple studies including *Nat Biotech* 2015, Wittrup et al., *Nature* 2012, Thurston et al. , and the

Duvall paper from 2019. The unique advantage of galectin-9 is in the sensitivity and fast kinetics making it ideal for high-speed live imaging. For screening and single time-point analysis galectin-8 and (and -3 as seen in Figure 1) is also appropriate. *In vivo* we believe sensitivity and specificity issues will be technique and tissue specific. Thus, the different galectins might find different niches within the field. Indeed, for staining of fixed tissues we mostly use galectin-3.

6. *Do the observe change in shape, size of the vesicles that show higher endosomal escape?*

Regarding size it is not really possible to draw conclusions using widefield microscopy. Regarding shape, we often see complex shapes after damage, though this is also hard to determine with live widefield microscopy. Using Airyscan we have confirmed these complex galectin patterns (see Supplementary Fig. 4c). Going forward this is one of our particular aims; to obtain higher resolution structures and gain functional and quantitative insights from these and not just anecdotal images, as is currently the case.

7. *Finally, I strongly support publication!! Well done!!*

We thank you for a thorough and highly constructive review.

Reviewer #2

This study supported lots of experiments to elucidate mechanisms of endosomal escape of cholesterol-conjugated siRNA induced by various endosome-disrupting small molecules. A large of data and relevant statistical analysis are provided in this paper. It is an interesting work and valuable for mechanism study on endosomal escape. However, there are still some confusions and list as following:

1. *The authors mentioned that galectins could respond to endosomal membrane disruption and relocate to damaged vesicles during lipid-mediated endosomal escape, in that way, can galectins be used as a sensor of membrane damage in other factors mediated endosomal escape as well? Or is there any other substance that can be used to probe endosomal escape?*

This is a highly relevant point. Currently it is not known which sensors are ideal for each class of delivery vehicle. In the present manuscript we show evidence for the utiliy of galectins for small-molecule facilitated release of a ligand conjugated siRNA. The use of galectins for lipid mediated release (*Nat Biotech* 2015, Wittrup et al.) and polymer mediated delivery (*e.g. ACS Nano* 2019, Duvall et al.) has also recently been demonstrated. Other potential sensors for release include *e.g.* the autophagy sensor LC3 as demonstrated in (*Nat Biotech* 2015, Wittrup et al.) however this sensor is downstream of the galectins and might not always be ideal. We have added a sentence on this lack of knowledge at present in the discussion section.

2. *Why choose cholesterol-conjugated siRNA instead of other siRNA nanocarrier delivery*

systems? How about the effects of small molecules induced endosomal escape on LNP-based or SNALP-based siRNA delivery?

Small molecule enhancers of LNP and SNALP delivery vehicles would also be of considerable interest. However, given the possibility to incorporate multiple functional elements on these larger delivery vehicles we currently believe that small-molecule adjuvants are particularly attractive for ligand conjugates. In addition, previous screens have shown that small-molecule enhancers of LNPs are completely different compounds than those improving cholesterol-siRNA knockdown and most probably acting through other mechanisms. This paper focused exclusively and in detail on endosomal escape enhancers for ligand-conjughated siRNA but in the future, we envision more detailed studies on other delivery vehicles will be pursued.

3. *Why choose siramesine and amitriptyline as membrane-destabilizing small molecules? Is there any other cationic small molecules also have the properties to facilitate endosomal escape?*

There are multiple drugs that have been shown to induce membrane damages. As stated in the introduction we chose to focus on the most extensively studied of these drugs. Given the finding that not all damages are equally efficient from an siRNA delivery perspective it will be highly interesting to perform focused screens of known membrane damaging compounds with regard to knockdown enhancement.

4. *What factors could possibly affect which kind of siRNA is accumulated in which types of intracellular compartments? For example, is it influenced by the sequence of siRNAs? Maybe by further understanding of this, we can better solve the mismatch between siRNA-containing and drug-targeting compartments which limits the siRNA activity improvement.*

This is a very interesting point and we believe a key to the success of future efforts to enhance ligand conjugated siRNA delivery. Indeed, how sorting, ligand-receptor interactions, dissociation of siRNA and ligand, recycling of receptors, and the sequence of siRNA influences endosomal escape most delivery vehicles is highly unclear. This will probably be an active research area within the field for many years, which we also aim to pursue. We have included an additional sentence on this aspect in the end of the discussion.

REVIEWERS' COMMENTS:

Reviewer #1 (Remarks to the Author):

Thanks to the authors for the thorough response. I support publication of this work.

Reviewer #2 (Remarks to the Author):

The authors have revised the manuscript and answered the questions according to my comments. Here is no more comments.

Reviewers' comments (2)

Reviewer #1 (Remarks to the Author)
Thanks to the authors for the thorough response. I support publication of this work.

Reviewer #2 (Remarks to the Author)
The authors have revised the manuscript and answered the questions according to my comments. Here is no more comments.

Response to reviewers' comments (2)

We are grateful to both referees for taking time to review this manuscript, and for providing valuable comments that helped improve the final paper. Thank you!
